# *Slc25a17* Gene Trapped Mice: PMP34 Plays a Role in the Peroxisomal Degradation of Phytanic and Pristanic Acid

**DOI:** 10.3389/fcell.2020.00144

**Published:** 2020-03-24

**Authors:** Paul P. Van Veldhoven, Evelyn de Schryver, Stephen G. Young, An Zwijsen, Marc Fransen, Marc Espeel, Myriam Baes, Elke Van Ael

**Affiliations:** ^1^LIPIT, Department of Cellular and Molecular Medicine, KU Leuven, Leuven, Belgium; ^2^Departments of Medicine and Human Genetics, David Geffen School of Medicine, University of California, Los Angeles, Los Angeles, CA, United States; ^3^Laboratory of Developmental Signaling, Department Human Genetics, VIB-KU Leuven, Leuven, Belgium; ^4^Department of Anatomy, Embryology, Histology and Medical Physics, Ghent University, Ghent, Belgium; ^5^Laboratory of Cell Metabolism, Faculty of Pharmaceutical Sciences, KU Leuven, Leuven, Belgium

**Keywords:** ATP, beta-oxidation, bile acids, coenzyme A, membrane transport, mitochondrial solute transporter, phytol, Refsum

## Abstract

Mice lacking PMP34, a peroxisomal membrane transporter encoded by *Slc25a17*, did not manifest any obvious phenotype on a Swiss Webster genetic background, even with various treatments designed to unmask impaired peroxisomal functioning. Peroxisomal α- and β-oxidation rates in PMP34 deficient fibroblasts or liver slices were not or only modestly affected and in bile, no abnormal bile acid intermediates were detected. Peroxisomal content of cofactors like CoA, ATP, NAD^+^, thiamine-pyrophosphate and pyridoxal-phosphate, based on direct or indirect data, appeared normal as were tissue plasmalogen and very long chain fatty acid levels. However, upon dietary phytol administration, the knockout mice displayed hepatomegaly, liver inflammation, and an induction of peroxisomal enzymes. This phenotype was partially mediated by PPARα. Hepatic triacylglycerols and cholesterylesters were elevated and both phytanic acid and pristanic acid accumulated in the liver lipids, in females to higher extent than in males. In addition, pristanic acid degradation products were detected, as wells as the CoA-esters of all these branched fatty acids. Hence, PMP34 is important for the degradation of phytanic/pristanic acid and/or export of their metabolites. Whether this is caused by a shortage of peroxisomal CoA affecting the intraperoxisomal formation of pristanoyl-CoA (and perhaps of phytanoyl-CoA), or the SCPx-catalyzed thiolytic cleavage during pristanic acid β-oxidation, could not be proven in this model, but the phytol-derived acyl-CoA profile is compatible with the latter possibility. On the other hand, the normal functioning of other peroxisomal pathways, and especially bile acid formation, seems to exclude severe transport problems or a shortage of CoA, and other cofactors like FAD, NAD(P)^+^, TPP. Based on our findings, PMP34 deficiency in humans is unlikely to be a life threatening condition but could cause elevated phytanic/pristanic acid levels in older adults.

## Introduction

Peroxisomes are important for β-oxidation of very long chain fatty acids, prostanoids, bile-acid intermediates and pristanic acid, for α-oxidation of phytanic acid and 2-hydroxy fatty acids and for synthesis of the precursors of ether lipids in mammals. These processes require a continuous flux of substrates, metabolites and cofactors across the peroxisomal membrane, the properties of which are still a matter of debate. *In vitro* data indicate that the membrane is leaky, a consequence of pore/channel activities (Van Veldhoven et al., 1987; Antonenkov et al., 2004; Antonenkov and Hiltunen, 2006). On the other hand, this membrane is claimed to be impermeable to (large) hydrophilic solutes such as nucleotides, at least *in vivo* (van Roermund et al., 1995; Henke et al., 1998). Based on the altered permeability of liver peroxisomes of a *Pxmp2*^–/–^ knock out mouse (Rokka et al., 2009), PMP22 allows entry of small neutral solutes (<300 Da) with an exclusion limit of about 600 Da. This implies the presence of specific or non-specific shuttle systems for transporting larger molecules. To date, only a few peroxisomal membrane proteins (PMP) involved in (solute) transport have been identified in mammals. Foremost are three PMPs belonging to the family of ATP-binding cassette (ABC) half transporters (Wanders et al., 2007). ABCD1, one of these ABC-transporters, previously called ALDP, causes X-linked adrenoleukodystrophy in man (Berger and Gartner, 2006) and plays a role in the peroxisomal uptake of very long chain fatty acids and/or their CoA-esters (van Roermund et al., 2008). ABCD3 was recently implicated in bile acid intermediates and pristanic acid degradation (Ferdinandusse et al., 2015).

Another mammalian peroxisomal transporter, PMP34, is a member of the mitochondrial solute carrier family (Wylin et al., 1998). This group of proteins is characterized by three tandem-repeated modules of approximately 100 amino acids, made up of two hydrophobic transmembrane α-helices joined by a large hydrophilic loop. Orthologs of PMP34, classified as member 17 of the solute carrier family 25 (SLC25A17), are found not only in mammals, but also in amphibians, fishes, insects, nematodes, yeasts and plants. In *C. boidinii* and *S. cerevisiae*, the (putative) orthologs PMP47 (Sakai et al., 1996) and ANT1 (van Roermund et al., 2001) respectively, facilitate the β-oxidation of medium-chain fatty acids (MCFA). In *Yarrowia lipolytica*, utilization of short chain alkanes (converted to short fatty acids) (Thevenieau et al., 2007) is dependent on this transporter. In plants, orthologs are involved in glyoxysomal β-oxidation during germination (Fukao et al., 2001; Arai et al., 2008; Linka et al., 2008) and the generation of auxins (Linka et al., 2008). In fact, *Arabidopsis* peroxisomes apparently contain at least three solute transporters: PMP38, considered to be the PMP34 ortholog (Fukao et al., 2001), and PNC1 and PNC2 (Linka et al., 2008). Only the latter ones are functional ATP/ADP + AMP counterexchangers.

The reports about other solute transporters in mammalian peroxisomes are a matter of debate. The association of Efinal, a calcium-binding ATP-Mg/phosphate exchanger (SLC25A24; SCaMC-1), with peroxisomes described in ileal enterocytes of rabbit (Weber et al., 1997), is questionable, given later reports revealing only a mitochondrial localization for SCL25A24, either by immunostaining of the endogenous protein in COS-7, HEK-293T, mouse 3T3-L1 or human fibroblasts, or upon expression of the human protein in COS-7 cells (del Arco and Satrùstegui, 2004). The claimed peroxisomal localization of a carnitine transporter (SLC22A3; OCTN3) (Lamhonwah et al., 2005) could not be confirmed in our lab, and more importantly, no peroxisomal abnormalities could be demonstrated in *Ocnt3*^–/–^ mice (Ansari S. and Van Veldhoven P.P., unpublished data). The putative presence of monocarboxylate transporters SLC16A1 (MCT1) (McClelland et al., 2003) and SLC16A7 (MCT2) (McClelland et al., 2003; Valenca et al., 2015) is only based on immunoblotting of purified rat liver peroxisomes (McClelland et al., 2003) or immunocolocalisation in prostate cancer cells (Valenca et al., 2015). Apparently, MCT2 is only partly associated with peroxisomes in malignant, but not in normal prostate cells (Valenca et al., 2015). Finally, except for PMP34, no other solute transporter was detected in proteomic studies on peroxisomes or peroxisomal membranes (Kikuchi et al., 2004; Wiese et al., 2007; Islinger et al., 2010; Gronemeyer et al., 2013).

Although human PMP34, similar to ANT1, can partially rescue the MCFA β-oxidation defect in *ant1*Δ yeast cells and is capable of transporting adenine nucleotides in an *in vitro* assay (Palmieri et al., 2001; Visser et al., 2002), its physiological role in mammalian peroxisomes remains unclear. In contrast to yeast, MCFA undergo β-oxidation in mitochondria and, as detailed below, ATP-dependent intra-peroxisomal reactions are scarcely documented in mammals. Very-long-chain acyl-CoA synthetase (ACSVL1; SLC27A2), containing a PTS1-like signal (LKL) in mouse and man, is thought to activate intra-peroxisomal pristanic acid produced via α-oxidation of phytanic acid (Steinberg et al., 1999). Phytanoyl-CoA hydroxylase, involved in α-oxidation, is stimulated by ATP (or GTP) (Croes et al., 2000), but likely this is limited to *in vitro* conditions. Furthermore, two ATP-consuming steps of cholesterol biosynthesis were reported to occur in peroxisomes (Olivier and Krisans, 2000), but the subcellular localization of the responsible enzymes is questionable (Ghys et al., 2002; Hogenboom et al., 2004a, b). Intraperoxisomal ATP might be required for proper folding of a subset of peroxisomal matrix proteins, as has been suggested by the fact that peroxisomal dihydroxyacetone synthase (DHAS) is misfolded in the absence of PMP47 in *C. boiidini* (Sakai et al., 1996). It is also possible that ATP is needed to remove damaged proteins since mammalian peroxisomes contain a protease resembling the mitochondrial Lon protease (Kikuchi et al., 2004). The human protease displays indeed an ATP-dependent peptidase activity (De Walque S. and Van Veldhoven P.P., unpublished data). Peroxisomes contain still other ATP-dependent proteins like the above mentioned ABC-transporters (Wanders et al., 2007) but their ATP-binding sites, however, face the cytosol. The same holds true for some ATP-dependent peroxins (Platta et al., 2008). Recent data on yeast and plant peroxisomal ABC-transporters, however, point to hydrolysis of acyl-CoA esters during their transport, implementing their reactivation in the peroxisomal lumen (van Roermund et al., 2012; De Marcos Lousa et al., 2013). How this mechanism can be applied to carboxylates that are only activated in the ER (e.g., dicarboxylic acids) in mammals was not addressed in these papers.

To gain insights into the role of PMP34 in mammals, we analyzed mice in which *Slc25a17*, the gene coding for PMP34, has been inactivated by insertional mutagenesis, with a focus on ATP-dependent processes^1^. During this work, Bernhardt et al. (2012) reported that the *Arabidopsis* PMP38 acted as a NAD^+^/AMP antiporter while Agrimi et al. (2012) showed that human PMP34 (SLC25A17) is a transporter for CoA, FAD, FMN and AMP, to lesser extent for ADP and NAD^+^, but almost not active on ATP, and functioning as a counter-exchanger (likely CoA, FAD, NAD^+^ inward; AMP outward). Intraperoxisomal CoA would be required for any activation step within these organelles, as already discussed above. In addition, it is a cofactor for the thiolytic cleavage in the β-oxidation cycle. Contrary to ATP, CoA is however generated inside the peroxisomal matrix via the action of acyl-CoA thioesterases, carnitine acyltransferases and conjugating enzymes (Hunt and Alexson, 2008; Van Veldhoven, 2010). In view of this new information, additional peroxisomal parameters were analyzed in the knock out mice. Phenotypic abnormalities in PMP34 deficient mice after phytol feeding point to a bottle neck in branched chain fatty acid degradation.

## Materials and Methods

### Materials

Radioactively labeled [1-^14^C]-hexadecanoic acid was obtained from PerkinElmer Life Sciences; 2-methyl-[1-^14^C]-hexadecanoic acid (Vanhove et al., 1991), 3-methyl-[1-^14^C]-hexadecanoic acid (Croes et al., 1996), [26-^14^C]-3,7,12-trihydroxycholestanoic acid (Casteels et al., 1988), [1,14-^14^C]-tetradecanedioic acid (Dirkx et al., 2007), [1-^14^C]-lignoceric acid (Vanhooren et al., 1994), 2-hydroxy-[1-^14^C]-octadecanoic acid (Foulon et al., 2005), and [2-^14^C]-6,9,12,15,18,21-tetracosahexaenoic acid (De Nys, 2002) were synthesized as described previously. The latter was repurified before use. Synthesis of pristanoyl-CoA (Van Veldhoven et al., 1991), phytanoyl-CoA (Van Veldhoven et al., 1996b) and 3-methyldodecanoyl-CoA (Croes et al., 2000) has been described before. Biotinylated azido-ATP analogs were obtained from Altbiosciences International. Primers and vectors used are listed in Supplementary Table S1.

### Mice Breeding and Manipulation

ES cells (E14Tg2a.4; derived from the 129/OlaHsd strain) were electroporated with a *Sal*I linearized gene trapping vector (pGT0pfs) by the BayGenomics consortium^2^. ES cells from a clone containing an insertional mutation in *Slc25a17* (clone XB686) were microinjected into host C57BL/6 blastocysts^3^. The resulting male chimeric mice were bred with C57BL/6 female mice; offspring heterozygous for the *Slc25a17* mutation were further backcrossed with C57BL/6 mice. During the initial intercrosses of heterozygous mice on a mixed genetic background (129/OlaHsd and C57BL/6) no knockout^4^ (KO) pups were born and the ratio of^±^versus ^+/+^ animals equaled 2.1, a significant departure from Mendelian ratios (*n* = 31; *P* < 0.001 by *c*^2^ statistics) (see Supplementary Material). With additional breeding onto a Swiss Webster (Taconic) background (5th and 8th generation), two *Slc25a17*^–/–^ pups were identified that survived into adulthood. In their progeny, no prenatal death of ^–/–^ embryos was seen. The reason for the embryonic lethality observed in the mixed genetic background is not fully understood, but this phenomenon is likely due to an unidentified mutation on a genetically linked “passenger” gene (see Supplementary Material).

Mice were kept in a temperature- and humidity-controlled facility with a 12-h light/dark cycle under specified pathogen-free conditions, with unlimited access to water and a standard enriched rodent chow (Trouw Nutrition France). At an age of 3-4 weeks, mice were ear-tagged and genotyped. Noon of the day on which the copulation plug was found was considered to be embryonic day 0.5 (E_0_._5_).

For some experiments, the standard chow was fortified with 0.3% (v/w) clofibrate (Fluka), 0.5% (w/w) phytol (Acros Organics), 0.5% (w/w) farnesol (Sigma-Aldrich), 1% (w/w) cholesterol (Acros Organics), 1.25% (w/w) cholesterol/0.5% (w/w) cholic acid (Acros Organics/Fluka), or 2% (w/w) cholestyramine resin (Sigma), or the drinking water was supplemented with 1% (v/v) ethylene glycol. In other experiments, mice were made diabetic by a tail vein injection with streptozotocin (1.5 mg/10 g body weight) or given a high fat chow (58 kcal% fat) containing 33.3% (w/w) coconut oil (D12331; Research Diets). All mouse experiments were approved by the local animal use committee.

During handling of the animals, motor coordination was monitored with a tail suspension test. More elaborated tests included hanging wire, inverted grid, and swimming test. Body composition of Avertin-sedated animals was obtained by whole body dual energy X-ray absorptiometry (DEXA) (PIXImus densitometer, Lunar Corp), and analysis of the data was performed by PixMus software.

MFP2 deficient (Baes et al., 2000) and liver specific *Pex5* knock out mice (Dirkx et al., 2005) were bred as described before.

### Histology

Tissues were isolated from freshly dissected anesthetized mice or from Nembutal-anesthetized mice perfused with 10% formalin via the heart, postfixed overnight in formalin at 4°C, and further processed/stained (Oil red O, immunocytochemistry) as described before (Hulshagen et al., 2008). For cytochemistry, cryostat sections of formaldehyde fixed livers were incubated with diaminobenzidine (DAB) and H_2_O_2_ in alkaline medium to reveal catalase activity (Roels et al., 1995). DAB-incubated sections were further processed for LM and EM sectioning according to standard methods. β-galactosidase staining of cryosectioned organs or fixed tissues/embryos was based on X-gal as substrate, modified from Sanes et al. (1986). Wild type tissues were always included as negative controls. When necessary, samples were post-fixed in formalin and processed for paraffin sectioning.

### Cell Culture

Mouse embryonic fibroblasts (MEF), prepared from E_12_._5__–__18_._5_ embryos by trypsinization of the skin, were cultured in Hepes buffered DMEM/F12 supplemented with antibiotics/antimycotic, Glutamax and 10% (v/v) FCS, and, to avoid growth crisis and polyploidy (Loo et al., 1987), immortalized by transfection with pRSV-SV40T before the 4th passage. For oxidation experiments, MEF were seeded in T25 flasks and incubated for 20-24 h with 4 μM ^14^C-labeled substrates (specific radioactivity ∼4.5 μCi/μmol, except for lignoceric acid, ∼20 μCi/μmol), bound to BSA (molar fatty acid/BSA ratio = 2), in Hepes buffered DMEM/F12 supplemented with 0.2% Ultroser G as described before (Van Veldhoven et al., 2001). Depending on the type of substrate, the amounts of ^14^CO_2_, ^14^C-formate, ^14^C-labeled acid soluble material and label incorporated into glycerolipids were determined.

For ATP measurements, immortalized MEF, seeded into T75 flasks, were transiently transfected with vectors encoding peroxisomally (pRSVL) or cytosolically (pGL3-CMV-luciferase) targeted luciferase using Lipofectamine Plus (Invitrogen). In intial experiments, cells were cotransfected with pCMVβ (Clontech), encoding cytosolic β-galactosidase. After 48 h, cells were trypsinized, washed and resuspended in modified Krebs-Ringer buffer. For each experimental condition, 10^6^ cells (in 100 μl) were transferred in triplicate to the wells of a white 96-well plate (Nunclon). For normalization, 0.5 × 10^6^ (in 50 μl) of resuspended cells were transferred in triplicate to wells containing 50 μl lysis buffer (1% Triton X-100 and 8 mM ATP in modified Krebs-Ringer buffer) and incubated at room temperature for 15 min. The plate was subsequently placed into a luminometer (Luminoskan Ascent, Labsystems) and 40 nmol luciferin was automatically injected into the wells (final assay volume 200 μl). Luminescence was monitored every ten sec for several min and only plateau values were considered. Values were normalized by dividing average plateau luminescence values in intact cells by average plateau luminescence values in lysed cells (representing total luminescence potential - the maximal luminescence that can be obtained in the presence of excess exogenous ATP in lysed transfected cells). Respective cytosolic and peroxisomal localizations of luciferase proteins encoded by pGL3-CMV-luciferase and pRSVL constructs were verified by immunocytochemistry in a separate experiment 48 h after transfection, using a commercial α-luciferase antibody (Promega) and FITC-conjugated rabbit α-goat antibody (Sigma).

To monitor NAD^+^ levels, cells were (co)electrotransfected (Neon Transfection System, Invitrogen) with pPVV311, encoding the catalytic domain of human poly-ADP ribose polymerase 1 (PARP1), amino acids 571-1014, preceeded by the FLAG-epitope and ending in KSKL. The insert of pPVV311 was generated by PCR on IMAGE clone 5193735 (Source Bioscience) using HsPARP1-1s-EP and HsPARP1-2r-XB as primers (Supplementary Table S1), restricted with *Pst*I and *Xho*I and subcloned into pCMVTag2B (Stratagene). Sequence was verified by LGC genomics. MEF were immunocytochemically analyzed 2 days post electroporation by fluorescence microscopy (Olympus IX-81 motorized inverted microscope equipped with a CCD-FV2T digital B/W camera) using α-PAR (mouse monoclonal antibody, clone 10H, Tulips Biolab) and rabbit α-PEX14 (Amery et al., 2000) or α-Flag M2 (rabbit polyclonal, Sigma) and mouse α-PEX14, followed by the appropriate Alexa Fluor 488- or TexasRed-labeled secondary antibodies (Invitrogen, Calbiochem). The mouse polyclonal anti-PEX14 was raised in C57BL/6 mice immunized with His_6_-tagged human PEX14 (Amery et al., 2000).

Mouse hepatocytes were prepared from livers of adult mice with a two-step perfusion technique as described (Dirkx et al., 2005) and seeded in collagen-coated T25 flasks and incubated for 6 h with 4 μM labeled substrates in 3 ml Williams E medium with supplements as described (Dirkx et al., 2005). Slices were prepared from freshly dissected liver as described (Mezzar et al., 2017) and incubated for 20 min in 0.5 ml Hanks medium containing ^14^C-carboxy labeled substrates bound to BSA (200 μM and molar fatty acid/BSA ratio 2, except for lignoceric acid, 80 μM and a ratio of 0.8).

### DNA and RNA Analysis

For genotyping, genomic DNA was extracted from ∼2 mm tail biopsies or ∼3 mm^2^ ear snips by a Hot-SHOT protocol (Truett et al., 2000) with some modification (replacing the Tris-HCl buffer with non-buffered Mops to neutralize the alkaline lysate). From the neutralized hydrolysate, 5 μl was used in a single 50 μl PCR reaction with 1.5 U Taq DNA Polymerase (Fermentas), 1xTaq buffer with KCl, 2 mM MgCl_2_, 0.4 mM dNTP and 0.5 μM of primer pairs [PMP34-in1-s-k/PMP34-in1-r-e, amplifying a region of intron 1 surrounding the pGT0pfs insertion site (413 bp); Gal-3s/Gal-3r, amplifying a part of the β-geo sequence of pGT0pfs (667 bp)]. The PCR program consisted of a 2 min denaturation step at 94°C followed by 34 cycles of 30 s at 94°C, 30 s at 60°C and 1 min at 72°C, ending with a 7 min incubation at 72°C (GeneAmp PCR System 2700, Applied Biosystems).

Total RNA was isolated from murine cells or tissues with Tripure (Roche). For Northern analysis, equal amounts of liver RNA (30 μg) were separated by agarose gel electrophoresis and blotted to Hybond N^+^ paper and probed with ^32^P-labeled *Pst*I fragment of plasmid pTW45 (Wylin et al., 1998), corresponding to base 395-827 of the *Slc25a17* open reading frame (924 bases in total).

For mRNA analysis of MEF, initial reverse transcriptase reaction (15 min at 23°C, 50 min at 42°C and 10 min at 95°C) was carried out in a GeneAmp PCR System 2700 (Applied Biosystems) on 500 ng RNA using 1x first strand buffer (Invitrogen), 2.5 μM random hexamers (New England Biolabs), 2.5 mM dNTP, 40 U RNase inhibitor (Rnasin, Promega), and 200 U M-MuLV enzyme (Invitrogen) in a final volume of 20 μl. Real time PCR reaction (2 min at 50°C, 10 min at 95°C for activation of AmpliTaq Gold DNA polymerase, followed by 40 cycles of 15 s at 95°C and 1 min at 60°C) was performed with a PRISM 7700 Sequence Detector (Applied Biosystems) in a 96-well microtiter plate (final reaction volume 20 μl). PMP34 real time PCR reactions were carried out in triplicate on 30 ng cDNA using primer pair PMP34-1 (1.13 μM each) (see Supplementary Table S1) and mastermix Plus for SYBR Green I (Eurogentec). The same reaction was carried out on plasmid pTW45 (10-fold serial dilutions containing 1-10^5^ copies of pTW45) to generate a standard curve. The ribosomal 18S RNA real time PCR reaction was performed in triplicate on 80 pg cDNA and on serial dilutions of cDNA using TaqMan Ribosomal RNA Control Reagents Kit (Applied Biosystems). Threshold cycles of unknown samples were converted to input amount of cDNA using these standard curves, as described in Applied Biosystems PRISM Sequence Detection System User Bulletin 2. Normalized PMP34 mRNA content of each sample was calculated by dividing the absolute PMP34 mRNA value by the 18S rRNA content. Controls without input cDNA were included in each run.

For hepatic mRNA analysis, initial reverse transcriptase reactions (5 min 65°C, 50 min 42°C and 15 mins 70°C) were carried out on 2 μg RNA using 1x first strand buffer (Invitrogen), 500 ng oligo(dT), 0.5 mM dNTP, 40 U RNase inhibitor (RNaseOUT^TM^, Invitrogen), 10 mM DTT and 200 U SuperScript^TM^ II enzyme (Gibco). Real time PCR reactions (20 min 95°C, followed by 40 cycles of 3 s at 95°C and 30 s at 60°C) were carried out in triplicate on 30 ng cDNA with appropriate primer pairs (see Table 1; 10-25 μM each; for PMP34 mRNA reaction, primer pair PMP34-2 was used), 10 μM TaqMan FAM (6-carboxyfluorescein used as reporter dye) - TAMRA (6-carboxy-tetramethyl rhodamine used as quencher dye) labeled probe (Eurogentec) and TaqMan Fast Universal PCR Master Mix (Applied Biosystems) in a 96-well microtiter plate (final volume 10 μl) in the 7500 Fast Real-Time PCR System (Applied Biosystems). Real time PCR reactions with β-actin primers and probe were carried out on each sample in triplicate in separate reaction vials. Gene expression levels were normalized to β-actin transcript levels. Controls without input cDNA were included in each experiment.

**TABLE 1:** Cofactor content of hepatic peroxisomes from control and *Slc25a17*^–/–^ mice.

	**Cofactor (pmol/mg protein)**			
**Genotype**	**Wild type**		**PMP34 knock out**	
Phytol	−	+	−	+
CoA	828	1310	896	1513
TPP	369	282	304	305

*Peroxisomes were purified from livers of single male wild type and age-matched PMP34 knockout mice fed a standard (−) or phytol (+) enriched diet and analyzed for CoA, TPP, and protein.*

FISH (Chaffanet et al., 1995) was performed on *Slc25a17^+/–^* MEF with an *Eco*RI-fragment of the pGT1.8 IRESβGeo(S) vector (base pairs 8-4897; identical to a portion of pGT0pfs) as a probe.

### Biochemical Analyses

Peroxisomes were purified from liver homogenates by differential centrifugation and subsequent Percoll and Nycodenz gradient centrifugation (Verheyden et al., 1992) and analyzed for CoA (Van Veldhoven and Mannaerts, 1986) and thiamine-pyrophosphate (Fraccascia et al., 2007). Membranes were isolated by means of carbonate treatment (Fujiki et al., 1982).

Most enzyme activities were measured in freshly prepared tissue homogenates according to published procedures: urate oxidase (Van Veldhoven et al., 1987), catalase (Van Veldhoven et al., 1996a), glucose-6-phosphatase (Van Veldhoven et al., 1996a), glutamate dehydrogenase (Van Veldhoven et al., 1996a), citrate synthase (Dirkx et al., 2005) and NADH-Coenzyme Q1 oxidoreductase (Complex I) (Dirkx et al., 2005). ACOX1 was measured with palmitoyl-CoA bound to albumin and N-ethylmaleimide pretreated samples (Van Veldhoven et al., 1992) and 4-aminoantipyrine/3-hydroxy-2,4,6-tribromobenzoate/peroxidase as coupling reaction (Van Veldhoven et al., 1997a). Although the assay employed does not allow discrimination between ACOX1 and ACOX3, the contribution of the latter in mouse liver is negligible, based on previous work (Van Veldhoven et al., 1994). MFP1 and MFP2 were measured photometrically with the appropriate isomers of 3-hydroxy-3-phenyl-propionyl-CoA (Baes et al., 2000). The activity of MFP1 was not corrected for the contribution of the mitochondrial enoyl-CoA hydratase to the dehydration of the L-isomer, estimated at 20% of the total L-specific dehydratase activity in livers of control mice.

Alanine glyoxylate aminotransferase activity was measured as follows: 10 μl purified liver peroxisomes (10-50 μg protein) were added to 40 μl prewarmed reaction mixture (0.1 M K-phosphate buffer pH 7.4, 0.04% (w/v) Thesit, 20 mM sodium glyoxylate, 40 μM pyridoxal phosphate, 0.1 M L-alanine) and incubated for 40 min at 37°C. The reaction was halted by adding 250 μl 10% (w/v) trichloroacetic acid followed by a centrifugation step (10 min 1500 g). The supernatant fluid (200 μl) was neutralized with 200 μl 1 M Tris base and 400 μl of assay mixture (0.1 M Tris-HCl pH 8.4, 0.5 mM NADH, 0.5 U lactate dehydrogenase) was added. After another 10 min of incubation at 37°C, absorbance was measured at 340 nm. The amount of pyruvate formed was calculated from the decrease in NADH and corrected for blank reactions, carried out in parallel omitting L-alanine.

β-galactosidase activity in tissue homogenates was measured at 575 nm by following the hydrolysis of 2 mM chlorophenol red β-D-galactoside in the presence of 10 mM DTT – 1 mM MgCl_2_ – 0.2 M Hepes pH 8.0 - 0.1% (w/v) Triton X-100 at 37°C (modified from Young et al., 1993).

For photoaffinity labeling, purified peroxisomes or membranes (max 100 μg protein) were added to Eppendorf tubes containing a cold reaction mixture (final concentrations 50 mM Tris-HCl pH 7.5, 0.1 mM EGTA, 0.5 mM orthovanadate, 10 mM NaF, 4 mM MgCl_2_ and 40 μM azido-biotin-ATP analog; 100 μl final). After exposure on ice for 2 min to UV-light (254 nm; Camag), samples were quenched with 5 μl 1 M dithiothreitol, followed by DOC/TCA precipitation, SDS-PAGE and blotting. Biotinylated proteins were visualized with avidin-technology (streptavidin-alkaline phosphatase, Sigma).

Commercial kits were used to measure bile acids in bile and feces (Sigma), and urinary oxalate (Trinity Biotech) and creatinine (Bioassay Systems). Blood glucose was measured using Glucocard memory strips (Menarini Benelux).

For lipid analysis, tissue samples (50-100 mg) were homogenized (Polytron homogenizer, Kinematica GmbH) in chloroform/methanol/water (2/1/0.8, v/v), followed by phase separation and washing. The lipid extracts were analyzed for plasmalogens by determining the acid-released aldehydes as phenylhydrazones by RP-HPLC (Foulon et al., 2005) and total phospholipids by phosphate measurements on ashed aliquots (Van Veldhoven and Mannaerts, 1987). Triacylglycerols, cholesterol and cholesterylesters were determined either in lipid extracts or after TLC, by coupled enzymatic assays as described before (Van Veldhoven et al., 1997b, 1998) except that cholesteryl esters and triacylglycerols were hydrolyzed chemically (0.5 M KOH in 90% (v/v) ethanol for 60 min at 75°C).

Total fatty acid profiles were obtained by hydrolyzing lipid extracts, dried in the presence of tricosanoic acid (C23:0) as internal standard (IS), in acetonitrile/HCl/water (900/43/57, v/v) (Aveldano and Horrocks, 1983). Fatty acids, recovered in hexane, were dried and silylated in 1% BDMCS-BDMSMTFA (Fluka)/pyridine (1/1, v/v; 1 h, 75°C) (Asselberghs S. and Van Veldhoven P.P., unpublished data). Derivatized fatty acids were subjected to GC-MS analysis [Thermo Finnigan TRACE GC-MS, Interscience; equipped with an automated cold-on-column injection device and an AT^TM^-5ms column (length 15 m, ID 0.25 mm, film thickness 0.25 μm) (Alltech)]. Gas flow 1.5 ml helium/min; ionization mode EI+; electron energy 35 eV; temperature program: hold 150°C 4 min, 6°C/min to 300°C, hold 300°C 10 min. Calculations of fatty acid concentrations were based on the internal standard and corrected for experimentally determined response factors for a calibrated fatty acid mixture.

Fatty acid composition of cholesteryl esters or triacylglycerols were analyzed by GC as follows: hexane extracts were prepared from approximately 10 nmol hydrolyzed cholesteryl esters or triacylglycerols, after acidification of the hydrolysis mixture with HCl. Extracts were dried in the presence of IS (C23:0), dissolved in methanol/HCl (95/5, v/v), and placed at 75°C for 1 h. The hexane-extracted fatty acid methyl esters (FAME) were dried, dissolved in 50 μl hexane and subjected to GC analysis (Varian 3800 GC), whereby 5 μl was splitless injected on a SPB^TM^ PUFA fused silica column (length 30 m, ID 0.25 mm, film thickness 0.2 μm) (Supelco, Bellfonte, United States) (FID; helium flow 1 ml/min; temperature program: hold 60°C 1 min, 10°C/min to 180°C, hold 180°C 4 min, 3°C/min to 230°C, hold 230°C for 20 mins). Calculations of fatty acid concentrations were based on the internal standard and known response factors.

Acyl-CoAs were extracted from liver tissue (∼100 mg) in the presence of 500 pmol internal standard, purified on 2-(2-pyridyl)ethyl-silica-SPE columns (Sigma), and converted into etheno-derivatives with chloroacetaldehyde which were separated by RP-HPLC (Waters Symmetry C18, 5 μm, 100Å, 4.6 × 150 mm column) and monitored with Waters 2475 Multi-wavelength Fluo-detector (ex/em 230/420) as described before (Mezzar et al., 2017). Signals were analyzed and integrated with the Breeze 3.20 software (Waters). Although excellent separation of straight chain acyl-CoA was obtained (using tetrabutylammonium sulfate as ionpair in the starting solvent to trap short chain acyl-CoAs and triethylamine in the eluting gradient to produce sharp peaks and to improve the elution of very long chain acyl-CoAs), some branched acyl-CoAs elute closely to straight chain acyl-CoAs. Various other RP-columns (with C18-phases from different companies or other RP-phases) and chromatographic conditions (solvents, ionpairing, temperature) were tested to obtain baseline separation but in vain, and tetrabutylammonium sulfate hampered coupling to mass spectrometry. For the internal standard, non-naturally occurring 3-methyldodecanoyl-CoA was selected which eluted in a clean region of the chromatogram, not only differently from the major straight chain acyl-CoAs but also from (unknown) phytol-metabolites.

Biliary bile acid profiles were monitored by LCMS. A 2 μl bile aliquot was diluted 100-fold in water, and mixed with an equal volume of acetonitrile containing 20 mM NH_4_-acetate and 50 μM 23-nor-3α,12α-dihydroxy-5β-cholanic acid (IS; Steraloids). The mixture was transferred at 10 μl/min to a 1100 LC/MSD Trap-VL (Agilent) equipped with ESI/MS-Vl and scanned for m/z 350-650 during 2 min in − and + mode.

### Immunological Procedures

Western blots were prepared according to standard procedures. Primary rabbit antibodies were directed against rat ACOX1 (Van Veldhoven et al., 1994), rat MFP2 (Novikov et al., 1994), rat thiolase (Antonenkov et al., 1997) and rat PEX14 (Fransen et al., 1998), We also used commercial rabbit antisera against bovine catalase (Rockland), human SLC25A17 (HPA060972; Atlas Antibodies), rat CYP4A (Affinity BioReagents), F4/80 (Serotec), bovine GDH (Rockland), and PAR (see above) and a monoclonal mouse anti-β-actin (Sigma).

## Results

### Targeting of the *Slc25a17* Locus

An insertional mutation in the *Slc25a17* locus in mouse ES cells was identified within the Baygenomics gene trap library (Stryke et al., 2003). The insertion, which contained an engrailed-2 (en2) splice acceptor site followed by a sequence coding for a promoter less β-geo cassette, results in a fusion transcript consisting of the sequences from the first exon of *Slc25a17* (54 coding bases) followed in frame by βgeo sequences (see Supplementary Figure S1A). Using a combination of primer walking within the first intron of *Slc25a17* (Supplementary Figure S1B) and DNA sequencing, the integration of pGT0pfs was identified (between base pairs 81,177,814 and 81,177,815 of the forward strand of chromosome 15; Ensemble release 45). This integration site corresponds to base pairs 10,117-10,118 of intron 1 of *Slc25a17*. The integration of the trapping construct into the mouse genome was verified by means of fluorescence *in situ* hybridisation (FISH) on *Slc25a17*^+/–^ fibroblasts. A single signal was observed on chromosome 15 (15E1) at the expected distance from the centromere (Supplementary Figure S1C). Heterozygous mice on the C57BL/6 and Swiss Webster genetic backgrounds were viable and exhibited normal fertility and vitality.

**FIGURE 1:**
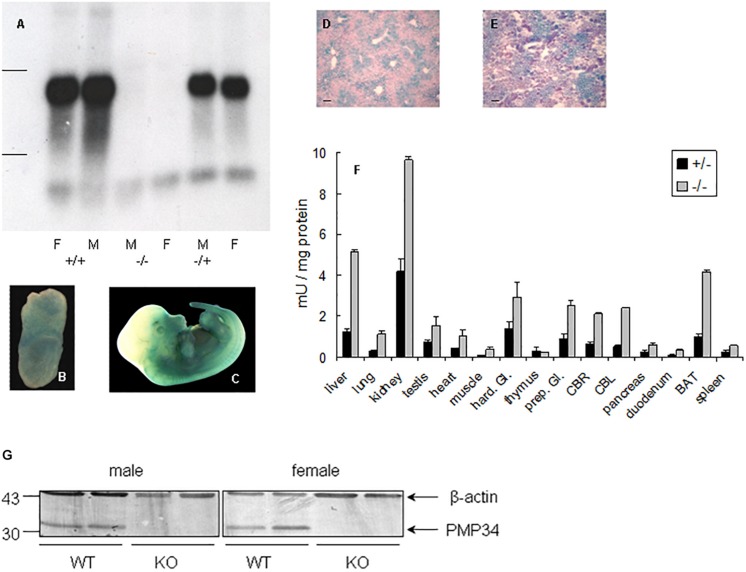
Expression of PMP34 in murine embryonic and adult life. **(A)** Northern analysis of RNA isolated from liver of age matched female (F) and male (M) mice of the indicated genotypes. Migration of ribosomal markers indicated at the left. **(B–E)** PMP34 expression in *Slc25a17*^+/–^ embryos at different prenatal stages or in adult mice as revealed by promoter-driven β-galactosidase reporter expression: whole mount staining at E_7_._5_ **(B)** and E_12_._5_ **(C)**; stained cryosections (10 μm) counterstained with Nuclear Fast Red from liver **(D)** and kidney **(E)** (Scale bar 10 μm; stainings of WT samples were negative). **(F)** β-galactosidase activity in tissues derived from 1 month old ^+/–^ male (black bars) or adult ^–/^male (gray bars). Values are represented as mean ± SD of separate measurements on two mice/genotype, except for liver (*n* = 5 for ^–/–^, *n* = 3 for ^+/–^). Galactosidase activities in tissues of WT mice were less then 0.05 mU/mg protein (not shown). Hard. Gl., Harderian gland; prep. Gl., preputial gland; CBR, cerebrum; CBL, cerebellum; duodenum, duodenal mucosa; BAT, brown adipose tissue. Panel **G**, PMP34 deficiency at the protein level. Total liver homogenates of adult wild type (WT) or PMP34-deficient (KO) mice, all containing equal amounts of protein, were processed for SDS-PAGE, Western blotting, and sequential immunoblot analysis with antisera specific for PMP34 and β-actin. The migration points of relevant molecular mass markers (expressed in kDa) are shown on the left. The arrows mark specific immunoreactive bands.

Northern analysis of liver RNA revealed absence of expression in ^–/–^ mice and no truncated mRNA species, starting from a secondary initiation site located behind the gene trap, were found^5^ (Figure 1A). Real time quantitative PCR on liver RNA provided similar information: PMP34 mRNA was half normal in the ^+/–^ and virtually absent^6^ in the ^–/–^ pups (Supplementary Figure S1E).

### Expression Profile of PMP34

Based on RT-PCR, PMP34 is ubiquitously expressed in mouse, with highest expression in liver, kidney and spleen (Wylin et al., 1998). Since expression of β-galactosidase in heterozygous (and KO) mice is under the control of the endogenous *Slc25a17* promotor, this reporter enzyme was used to study PMP34 expression. Expression was negligible at E_2_._5_ and E_3_._5_, but ubiquitous from E_6_._5_ to E_8_._5_. At later stages, staining was most prominent in the ventral area (Figures 1B,C). In adult mice, the PMP34 promotor was most active in kidney, followed by liver (with a zonal expression) and brown adipose tissue, compatible with a role in fatty acid oxidation (Figures 1D–F and Supplementary Figure S2). Various attempts, either by companies or in the lab, to generate a highly specific and sensitive antiserum against synthetic peptides (*Mm*PMP34_127__–__141_) or recombinant fusions (His_6_-*Hs*PMP34_1__–__60_, His_6_-*Hs*PMP34_129__–__204_) were unsuccessful (Van Ael E., Brees C., Fransen M. and Van Veldhoven P.P., unpublished data) preventing expression profiling at the protein level. However, very recently, we identified a commercial antiserum able to recognize PMP34 in whole liver homogenates (Figure 1G).

### Phenotypic Characterization of PMP34 Knockout Mice

Intercrossing of heterozygous mice yielded offspring in the expected Mendelian ratios for both genders (number of ^+/+^, ^+/–^ and ^–/–^ pups, derived from 24 nests: 34/59/34 for females, 30/66/27 for males). All PMP34 KO mice survived into adulthood and lacked any obvious phenotype, with normal weight, appearance, gross organ morphology, and motor coordination. Both males and females were fertile.

**FIGURE 2:**
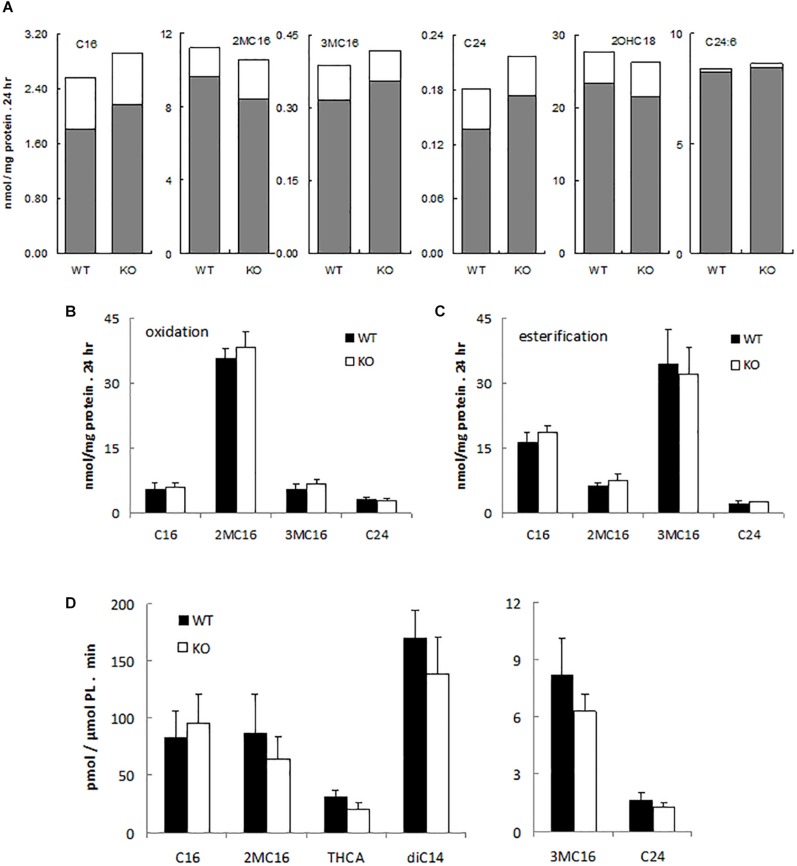
α- and β-oxidation in PMP34 deficient cells. Primary MEF **(A)**, immortalized MEF **(B,C)** or liver slices **(D)** from wild type (WT) and PMP34 knockout (KO) embryos or mice were analyzed for α- and β-oxidation using ^14^C-labeled substrates. Data for primary MEF **(A)**, derived from a single WT or KO E14.5 embryo (C57BL/6 background), are based on single incubations at 310-430 μg protein/T25 falcon, followed by analysis of labeled oxidation products (white bars: CO_2_; gray bars, acid soluble material or formate in case of 3-methylbranched fatty acids), normalized to protein content of the monolayer. Incorporation of fatty acids in glycerolipids and total uptake was comparable between the genotypes (not shown). Similar findings were obtained when incubations were done at other cell densities. Data for immortalized fibroblasts **(B,C)**, derived from 2 different embryos per genotype, are based on duplicate incubations at 110-180 μg protein/T25 falcon, and expressed as mean ± SD. Oxidation **(B)**, normalized to protein, represent the sum of CO_2_ and acid soluble material or to CO_2_ plus formate in case of 3-methyl-branched fatty acids. Total uptake by fibroblasts, calculated as oxidation plus esterification, the latter representing the label recovered in cholesterylesters, triacylglycerols and phospholipids **(C)**, was not different between WT and KO cells. Data for liver slices **(D)** are based on duplicate incubations with slices derived from 3 male animals per genotype and represent the sum of CO_2_ and acid soluble material or CO_2_ plus formate in case of 3-methyl-branched fatty acids, expressed as mean ± SD (normalized to phospholipids, PL). For no substrate, differences in CO_2_ or acid soluble material production were statistically significant except for THCA (*P < 0.05). Esterification rates for fatty acids were comparable (not shown). Dicarboxylic acids, 2-hydroxy long chain fatty acids, and bile acids are hardly or not incorporated into triacylglycerols and phospholipids, hence esterification of these substrates was not analyzed. C16, [1-^14^C]-hexadecanoic acid; 2MC16, 2-methyl-[1-^14^C]-hexadecanoic acid; 3MC16, 3-methyl-[1-^14^C]-hexadecanoic acid; C24, [1-^14^C]-tetracosanoic acid; 2OHC18, 2-hydroxy-[1-^14^C]-octadecanoic acid; C24:6, [2-^14^C]-6,9,12,15,18,21-tetracosahexaenoic acid; THCA, [26-^14^C]-3,7,12-trihydroxycholestanoic acid; diC14, [1,14-^14^C]-tetradecanedioic acid.

To assess possible peroxisomal abnormalities in PMP34-deficient mice, fatty acid oxidation was analyzed in fibroblasts. All peroxisomal β-oxidation substrates tested (lignoceric acid; 2-methylhexadecanoic acid, a pristanic acid analog; and 6,9,12,15,18,21-tetracosahexaenoic acid, a precursor of docosahexaenoic acid (DHA; C22:6) (De Nys, 2002) were degraded by primary ^–/–^ MEF (Figure 2A) and immortalized ^–/–^ MEF (Figure 2B), and rates were comparable to those seen in wild type fibroblasts. Likewise the α-oxidation of 3-methylhexadecanoic acid, a phytanic acid analog, and of 2-hydroxyoctadecanoic acid was not impaired (Figures 2A,B).

Similarly, all these fatty acids were oxidized by primary PMP34 deficient hepatocytes and additionally, the degradation of dicarboxylic acids (tetradecanedioic acid) and bile acid intermediates (trihydroxycholestanoic acid, THCA) appeared to proceed almost normal (data not shown). To avoid uncertainties with regard to contribution of dead adherent cells to the normalizing parameter (DNA or phospholipids), used in the hepatocytes experiments, hepatic slices were tested. Again, no clear deficiency was revealed, and only THCA oxidation was significantly reduced (∼30%) (Figure 2D). The lower activity with THCA was mainly due to less CO_2_ production (data not shown).

To evaluate a possible effect on etherphospholipid synthesis, plasmalogens were analyzed. The levels in heart, brain and liver were normal in the PMP34 KO mouse (see Supplementary Table S2). Likewise, no differences were observed in hepatic levels of cholesterol, cholesteryl esters, triacylglycerols, arachidonic acid (C20:4), DHA and lignoceric acid (data not shown; phytanic acid and pristanic acid were below detection limit). Additionally, MS analysis of bile revealed no abnormal bile acids or accumulation of bile acid intermediates (data not shown; see further).

Because peroxisome deficiencies often result in hepatic abnormalities (Baes and Van Veldhoven, 2016), this organ was examined in more detail. In first instance, the possibility of PPARα activation, a phenomenon observed in some mouse models with peroxisomal deficiencies (Fan et al., 1998; Baes et al., 2000), was examined. Expression of PPARα target genes, coding for multifunctional protein 1 (MFP1, encoded by *Ehhadh*), acyl-CoA oxidase 1 (ACOX1), cytochrome P450, family 4, subfamily a, polypeptide 10 (CYP4A10) and carnitine palmitoyltransferase1-β (CPT1-β)^7^ were analyzed by qPCR but no changes were observed in the PMP34 KO mice (Supplementary Figure S3). There was some variability in CYP4A expression but this was expected as the expression of members of the CYP450 superfamily is known to be variable (Kirby et al., 1993). Using a combination of immunohistochemistry, Western blot and activity measurements, the hepatic expression of peroxisomal, mitochondrial and ER proteins was investigated in the PMP34 KO mice. No specific abnormalities were detected in the expression and/or levels of the peroxisomal marker enzyme catalase, peroxins PEX13 and PEX14, key β-oxidation enzymes ACOX1, MFP1, MFP2 (encoded by *Hsd17b4*), and ACAA1 thiolase, peroxisomal membrane protein ABCD3 (PMP70), mitochondrial glutamate dehydrogenase (GDH) or CYP4A (results not shown). SDS-PAGE analyis of purified liver peroxisomes and their membranes revealed no clear-cut differences in protein composition between KO and wild type mice (Supplementary Figure S4). PMP34 is indeed a very minor PMP (Van Veldhoven and Mannaerts, 1994).

### Peroxisomal Cofactor Content

Based on indirect evidence, peroxisomal content of FAD, NAD^+^ and CoA (comparable β-oxidation rates for most substrates, see above) and of NADPH [comparable C22:6 levels that depend on peroxisomal 2,4-dienoyl-CoA reductase (DECR2) activitiy (De Nys et al., 2001)] is not considerably reduced in the absence of PMP34. To demonstrate abnormalities in the PMP34-deficient mice, the peroxisomal cofactor content was investigated in a more direct approach. We first focused on CoA (Van Veldhoven and Mannaerts, 1986) and thiamine pyrophosphate (TPP) (Fraccascia et al., 2007), shown previously to be measurable in isolated peroxisomes. CoA and TPP contents of hepatic peroxisomes purified from the KO and wild type mice were comparable (Table 1). As older studies suggested that hepatic peroxisomes leak most of their ATP and NAD^+^ during isolation (Mannaerts et al., 1982; Van Veldhoven et al., 1987), the measurement of these cofactors required a different approach. Firstly, biotinylated photoaffinity ATP-analogs were used to reveal ATP-binding proteins in isolated peroxisomes and their membranes. Although some proteins could be visualized, this approach failed to label a protein of the expected size or to show a difference in membranes purified from WT and PMP34 KO peroxisomes (Supplementary Figure S4; Meyhi E. and Van Veldhoven P.P., data not shown). Secondly, we employed peroxisomally (and cytosolically) targeted firefly luciferase to probe the peroxisomal (and cytosolic) ATP content of immortalized PMP34 KO fibroblasts *in vivo*. Others have shown that the amount of luminescence, generated by luciferase in the presence of luciferin, is a measure for ATP (Strehler, 1968) and that this principle can also be applied to luciferase targeted to different subcellular compartments (Jouaville et al., 1999; Manfredi et al., 2002). The correct targeting of the peroxisomal luciferase was verified by immunocytochemistry. Using this approach to probe peroxisomal and cytosolic ATP in immortalized fibroblasts, we could not measure any differences between the *Slc25a17*^–/–^ and ^+/+^ cells (Supplementary Figure S5).

To visualize the peroxisomal NAD^+^-pool, we expressed a peroxisomally targeted catalytic domain of poly-ADP ribose polymerase (PARP-SKL) in MEF. According to Dölle et al. (2010), such fusion acts as a molecular detector of local NAD^+^, converting it into poly-ADP-ribose (PAR). In cells expressing PARP-SKL, presence of PAR in peroxisomes was revealed in both genotypes. The amount of peroxisomal PAR in the ^–/–^ cells, relative to the amount of PARP-SKL, appeared to be higher than in the ^+/+^ cells (Supplementary Figure S6).

To glean information on the peroxisomal pyridoxal-phosphate status, mice were given ethylene glycol. Ethylene glycol is converted via glycolate to glyoxylate, which is removed by the peroxisomal alanine-glyoxylate aminotransferase (AGT). Insufficient AGT activity leads to further oxidation of glyoxylate to oxalate, which causes renal insufficiency (Salido et al., 2006). Since AGT activity is dependent on pyridoxal-phosphate, dietary ethylene glycol supplementation could unveil a defect in the transport of this cofactor into peroxisomes. Treatment with ethylene glycol increased urinary oxalate levels, but there was no marked difference between the genotypes in general appearance, gross organ morphology, or in hematoxylin/eosin stained liver or kidney sections (results not shown). In addition, AGT activity measured in isolated liver peroxisomes in the absence of pyridoxal-phosphate did not differ between (non-treated) wild-type and KO mice and equaled the values obtained in the presence of externally added pyridoxal-phosphate, indicating that transport and./or content of pyridoxal-phosphate is unaffected by the absence of PMP34 (Supplementary Figure S7).

### Challenging of Adult PMP34 Deficient Mice

Given the absence of an obvious phenotype in the KO animals, mice were exposed to experimental conditions that are believed to increase flux through peroxisomal pathways. Thus mice were fasted, treated with Clofibrate, or given a diet enriched in farnesol, cholesterol, or phytol. In addition, mice were administered cholesterylamine resin, a coconut oil diet or made diabetic. Except for phytol, none of these treatments affected the PMP34 KO and control mice differentially (see Supplementary Material).

**FIGURE 3:**
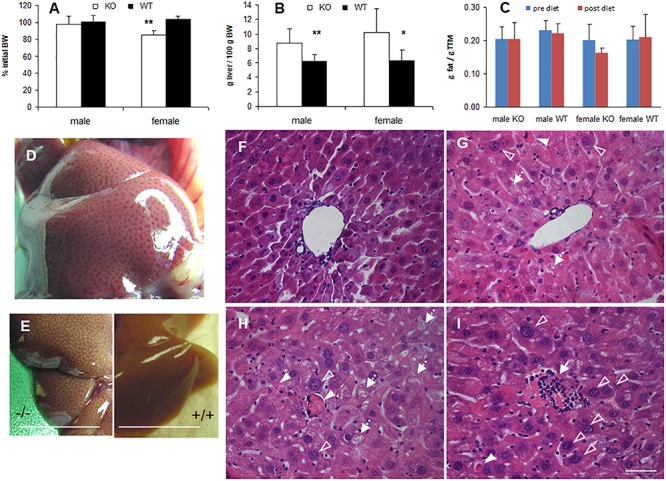
Phenotype of phytol challenged PMP34 knockout mice. **(A–C)** Influence of phytol diet on male or female PMP34 knock out (KO) mice versus age matched wild type (WT) mice on body weight, expressed as % of body weight (BW) at the start of the diet **(A)**, liver size, expressed in g/100 g BW **(B)**, and fat mass, expressed in g/g total tissue mass (TTM) as determined by DEXA at the start and end of the diet **(C)**. Data are represented as mean ± SD (*n* = 4-8 **(A,B)** or 3-6 **(C)** per gender and per genotype. The effect of phytol was statistically analyzed per gender (Oneway ANOVA **P < 0.01, *P < 0.05 KO versus WT). Heterozygous mice responded in a similar way to phytol diet as WT animals and liver size and fat mass of KO and WT did not differ on a control diet (data not shown). **(D,E)** Mottled appearance of liver of knockout mice on a phytol diet, as seen upon dissection of a male mouse **(D)** or after bleeding and removal of the liver (**E**; scale bar 1 cm). Similar mottling is evident in female KO mice (not shown). **(F–I)** H&E stained liver sections of WT **(F)** and KO male mice **(G–I)** on phytol enriched diet showing enlarged nuclei (open arrowhead), apoptotic bodies (full arrowhead), inflammation zones (arrow) and microvesicular steatosis (dashed arrow) in the phytol-fed KO mice. **(F,G)**: portal triads; **(H,I)**: intermediary zone of the hepatic lobule. Scale bar 50 μm.

**FIGURE 4:**
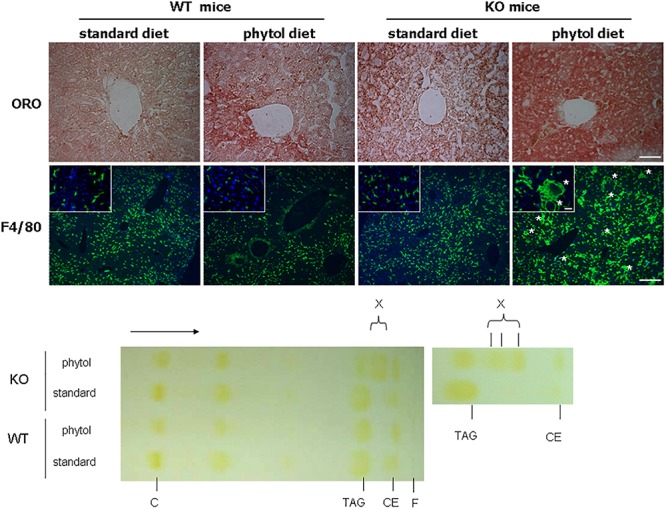
Hepatic steatosis in phytol challenged PMP34 knockout mice. Liver sections of male control (WT) and knockout (KO) mice fed standard or phytol enriched diet were stained with Oil red O (ORO) (**top** row; scale bar 50 μm) or immunostained for macrophage marker F4/80 plus DAPI (second row; scale bar 200 μm; inset scale bar 25 μm), showing in the liver of the KO mice fed phytol accumulation of neutral lipids or enlarged macrophages in the process of phagocytosing cell debris (asterisks). **Bottom**, left: representative TLC of lipid extracts of liver of male age-matched WT and KO mice fed standard or phytol diet, visualized by iodine staining (solvent hexane/diethylether/acetic acid 70/30/1, v/v; portion of plate shown; direction of solvent indicated by arrow and its front by F). Standards, migration indicated, were cholesterol (C), triolein (triacylglycerides, TAG), cholesteryloleate (cholesterylester, CE); X indicates presence of new TAG species. When using hexane/diethylether/acetic acid (80/20/1, v/v) as solvent, spot X is splitting up in separate bands (right panel).

In many animal studies, phytol is used as a precursor of phytanic acid, which is broken down in peroxisomes by sequential α- and β-oxidation reactions (Gloerich et al., 2007; Van Veldhoven, 2010). When male or female adult mice were fed a phytol-rich diet (0.5% (w/w), 20-30 days), the livers of KO mice were enlarged and had a mottled appearance (Figures 3B,D,E). No additional differences in gross morphology of other organs were observed. Females, however, tend to loose body weight and adipose tissue (Figures 3A,C). Hematoxylin/eosin staining revealed an acute hepatic inflammatory reaction in the KO mice, but not in the control mice (Figure 3F). The phytol-fed KO mice exhibited hepatic inflammation zones, apoptotic bodies, enlarged and smaller nuclei and microvesicular steatosis (Figures 3G–I). To better document the hepatic steatosis, liver sections were stained with Oil red O, revealing an accumulation of neutral lipids in the livers of the phytol-fed KO mice, which was much more intense than in the wild type mice on the same diet (Figure 4). Other organs or tissues (kidney, testis, spine, brain) had no visible lipid accumulation (data not shown). Staining for the macrophage marker F4/80 revealed in the liver of phytol-treated KO mice enlarged Kupfer cells that appeared engulfing cellular debris (Figure 4).

Because phytol causes hepatic peroxisome proliferation and induces many genes involved in lipid and glucose metabolism in rodents, we examined some lipid-related genes in phytol-fed mice. MFP1, ACOX1, CYP4A10 and CPT1-β upregulation was more pronounced in the phytol-fed PMP34 KO mice than in the wild type mice (Supplementary Figure S8A). Hepatic protein levels were investigated by enzymatic activity measurements, western blot and immunohistochemistry. Based on immunohistochemical data, ACOX1 and CYP4A were induced to a greater degree in the livers of the phytol-fed KO mice than in wild type mice (Supplementary Figure S8C). Furthermore, the peroxisomal proteins catalase and PEX14 were also induced to a higher degree in the challenged KO mice (Supplementary Figure S8C). Surprisingly, the increased abundance of catalase and ACOX1 observed by immunohistochemistry was not mirrored by increased enzymatic activities of these proteins (Supplementary Figure S8B), or immunosignals on blots (data not shown). Only in case of MFP1, the elevation of enzyme activity was more pronounced in the challenged KO mice than in the wild-type mice on the phytol diet.

**FIGURE 5:**
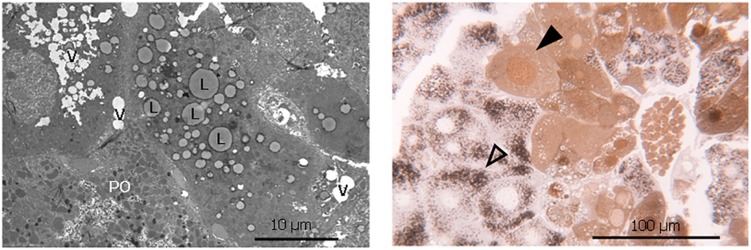
Lipid accumulation, vacuolization and dual hepatocyte population in liver of phytol challenged PMP34 knockout mice. **Left panel:** Electron microscopic image of a liver section from an adult male phytol fed PMP34 knockout mouse, stained for catalase and contrast stained with uranyl acetate and lead citrate. Lipid accumulation (L) and vacuolization (V) are clearly visible in the hepatocyte lacking catalase positive peroxisomes, adjacent to a hepatocyte containing DAB-reactive peroxisomes (PO). **Right panel:** Light microscopic image of a semithin liver section from an adult male phytol fed PMP34 knockout mouse. A dual population of hepatocytes can be observed: aberrant hepatocytes, lacking DAB-reactive peroxisomes and containing cytoplasmic and nuclear catalase (closed arrowhead) are discernible adjacent to normal hepatocytes containing DAB-reactive peroxisomes (open arrowhead).

The increased lipid accumulation and vacuolization in the livers of the phytol fed PMP34 KO mice was clearly confirmed with electron microscopy (Figure 5). Strikingly, a dual population of hepatocytes was observed in the phytol fed PMP34 KO mice: besides hepatocytes in which the presence of proliferated peroxisomes was evident, in other cells (depending on the area investigated up to 30%), catalase containing peroxisomes were absent and a cytoplasmic localization of catalase was seen, which was confirmed using light microscopy (Figure 5). In many of these latter cells, catalase was also present in the nucleus. The incidence of such hepatocytes was much lower in the condition of the phytol fed wild type mice (estimated at maximum 5%).

**FIGURE 6:**
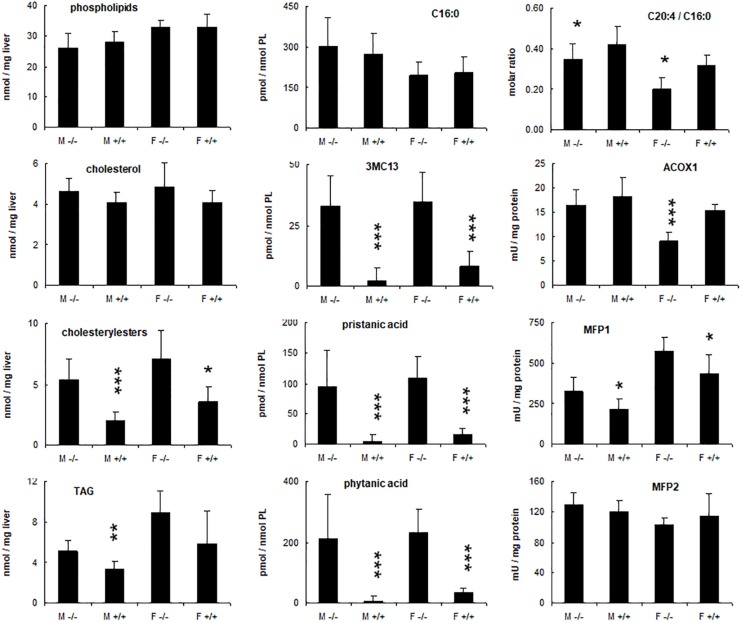
Hepatic changes in phytol treated PMP34 knockout mice. Livers of adult male (M) or female (F) wild type (+/+) or knockout mice (–/–) given a 0.5% phytol enriched diet for 3 weeks were analyzed for protein and lipid content, fatty acid composition, and peroxisomal β-oxidation enzymes. Small amounts of branched fatty acids were present in all +/+ females, but only in one +/+ male animal detected (hence larger SD), whereas severe but variable accumulation was seen in all –/– animals. Arachidonic acid (C20:4) was expressed relative to the amount of palmitic acid (C16:0) per liver. ACOX1 values are several fold higher than in animals receiving a normal diet (see Supplementary Figure S3). Values are mean ± SD for 4-6 animals per gender and per genotype, treated in four different feeding studies. Values in heterozygous mice were similar to those in wild type animals (data not shown). Statistical differences are calculated per gender (Oneway ANOVA ***P < 0.005, **P < 0.01, *P < 0.05 KO versus WT). TAG, triacylglycerides; PL, phospholipid; 3MC13, 4,8,12-trimethyltridecanoic acid; mU, nmol/min.

Given these alterations, the nature of the accumulating lipids was investigated. Consistent with the Oil red O staining, cholesteryl ester and triacylglycerol levels were increased in the livers of KO mice fed phytol as compared to the control mice on this diet (Figure 6). For the triacylglycerols, this was mainly due to the presence of novel species with a distinctive TLC migration pattern (higher R_*f*_) (Figure 4). Similar species have been reported in cardiac triacylglycerols of rabbits fed phytol (Sezille et al., 1970), and mice lacking HACL1 when exposed to phytol (Mezzar et al., 2017). GC analysis showed that both cholesteryl esters and triacylglycerols were enriched in phytanic and pristanic acid, although with a different ratio^8^.

**FIGURE 7:**
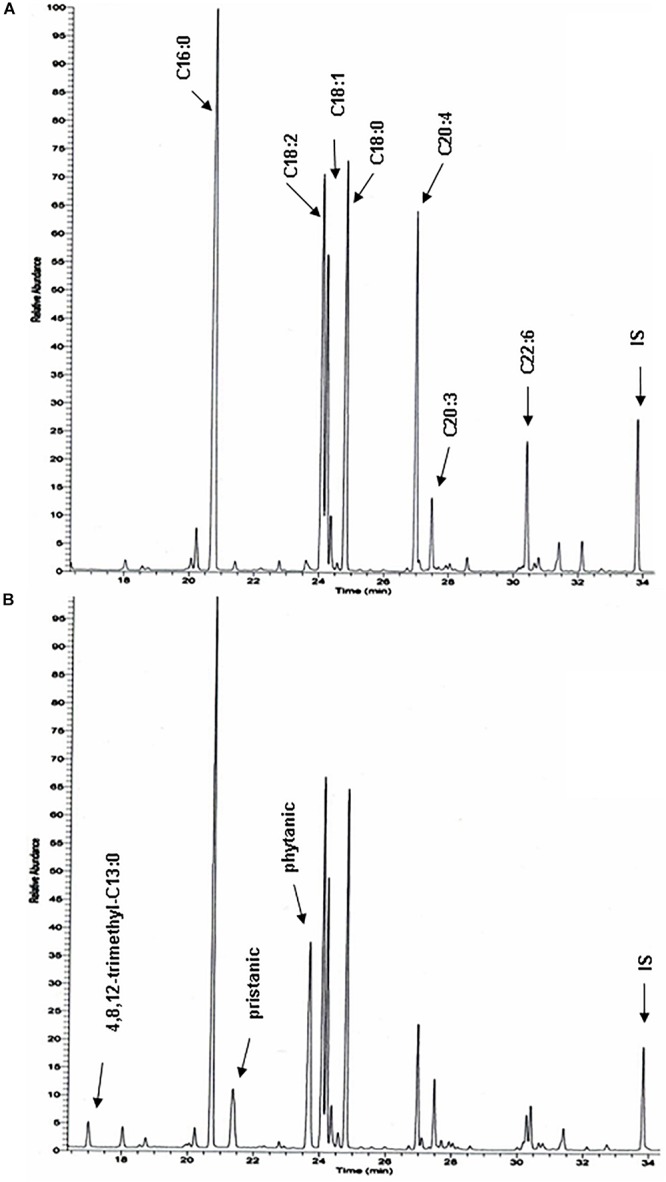
Total hepatic fatty acid profiles of phytol challenged PMP34 knockout mice. GC-MS profiles of tert-butyldimethylsilyl esters prepared from total lipid extracts from livers of age-matched male wild type **(A)** and knockout **(B)** mice fed phytol diet. A portion of the chromatogram (15-35 min) is shown. Major peaks are labeled for the wild type sample **(A)**, and extra peaks appearing in the knockout sample are indicated in panel B. Using a temperature program better suited for separation of medium chain fatty acids, 2,6,10-trimethylundecanoic acid is also seen in the KO profiles (not shown) (IS, internal standard; tricosanoic acid, C23:0).

To document changes in fatty acids which are normally not incorporated into neutral glycerolipids (such as polyunsaturated fatty acids, PUFA), fatty acid composition of total liver lipid extracts was analyzed. Consistent with the triacylglycerol changes described above, phytanic and pristanic acid were among the major fatty acids in the livers of the phytol fed KO mice (Figure 7). In females, presumably due to lower SCPx levels (Atshaves et al., 2004), the levels were consistingly higher than in males (Figure 6). Interestingly, pristanic acid degradation products like 4,8,12-trimethyltridecanoic and 2,6,10-trimethylundecanoic acid, were noticed upon closer analysis of the GC-MS profiles of the KO extracts (Figures 6, 7; data not shown). In addition, this analysis revealed that arachidonic acid and DHA were reduced in the livers of the KO mice fed phytol as compared to the wild type mice on this diet (Figures 6, 7; data not shown). The levels of hepatic very long chain fatty acids (C24:0) however, remained normal (data not shown).

**FIGURE 8:**
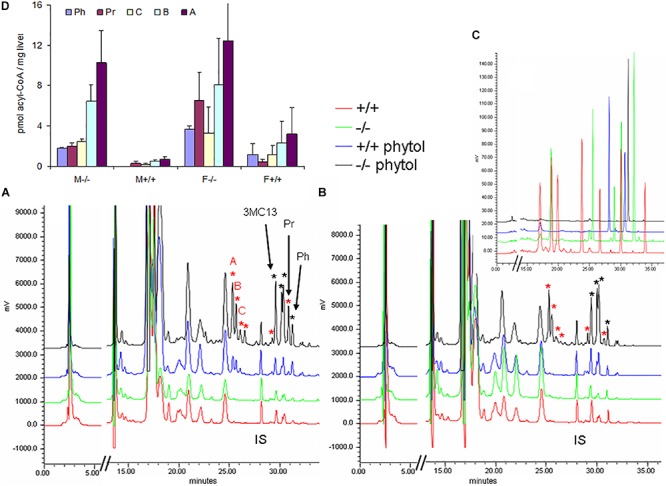
Hepatic acyl-CoA profiles of phytol challenged PMP34 knockout mice. Panels **(A,B)** show representative RP-chromatograms of etheno-acyl-CoAs (and other adenine-containing compounds), prepared from liver extracts of female **(A)** or male **(B)** wild type and PMP34 deficient mice on a normal or phytol-enriched diet, spiked with internal standard (500 pmol 3-methyldodecanoyl-CoA, IS). Arrows indicate the elution, based on spiking and co-elution, of phytanoyl-CoA (Ph), pristanoyl-CoA (Pr), and 4,8,12-trimethyltridecanoyl-CoA (3MC13). New peaks appearing in the phytol-treated female +/+ animals, and further increased in all –/– mice, are marked with a red asterisk. Some phytol-derived intermediates, marked with a black asterisk, elute close to straight acyl-CoAs, impeding baseline separation. The elution of different standards as etheno-derivatives, is shown in panel C (taken from Mezzar et al., 2017): mix 1 (red) contains CoA, acetyl-, propionyl-, hexanoyl-, decanoyl-, palmitoyl- and lignoceroyl-CoA; mix 2 (green) acetyl-, octanoyl-, tetradecanoyl-, palmitoyl- and eicosanoyl-CoA; mix 3 (blue), IS and pristanoyl-CoA; mix 4 (black) phytanoyl-CoA. Panel **(D)** shows the quantification of known and some unknown, labeled as **A–C** in panel **(A)**, phytol-derived acyl-CoAs (values based on 2 separate mice per gender and per genotype; mean ± SD).

Hepatic acyl-CoA profiling showed various additional acyl-CoA-esters in the phytol-treated PMP34 deficient mice, including phytanoyl-CoA and pristanoyl-CoA (Figures 8A,B). Due to lack of standards, not all accumulating CoA-esters could be identified, but one co-eluted with the CoA-esters of the above mentioned 4,8,12-trimethyltridecanoic acid. Compared to the amount of phytol-derived branched chain fatty acids incorporated in hepatic lipids, their CoA-esters represent a minor fraction (1/1000-1/2000), as expected for catabolic intermediates.

**FIGURE 9:**
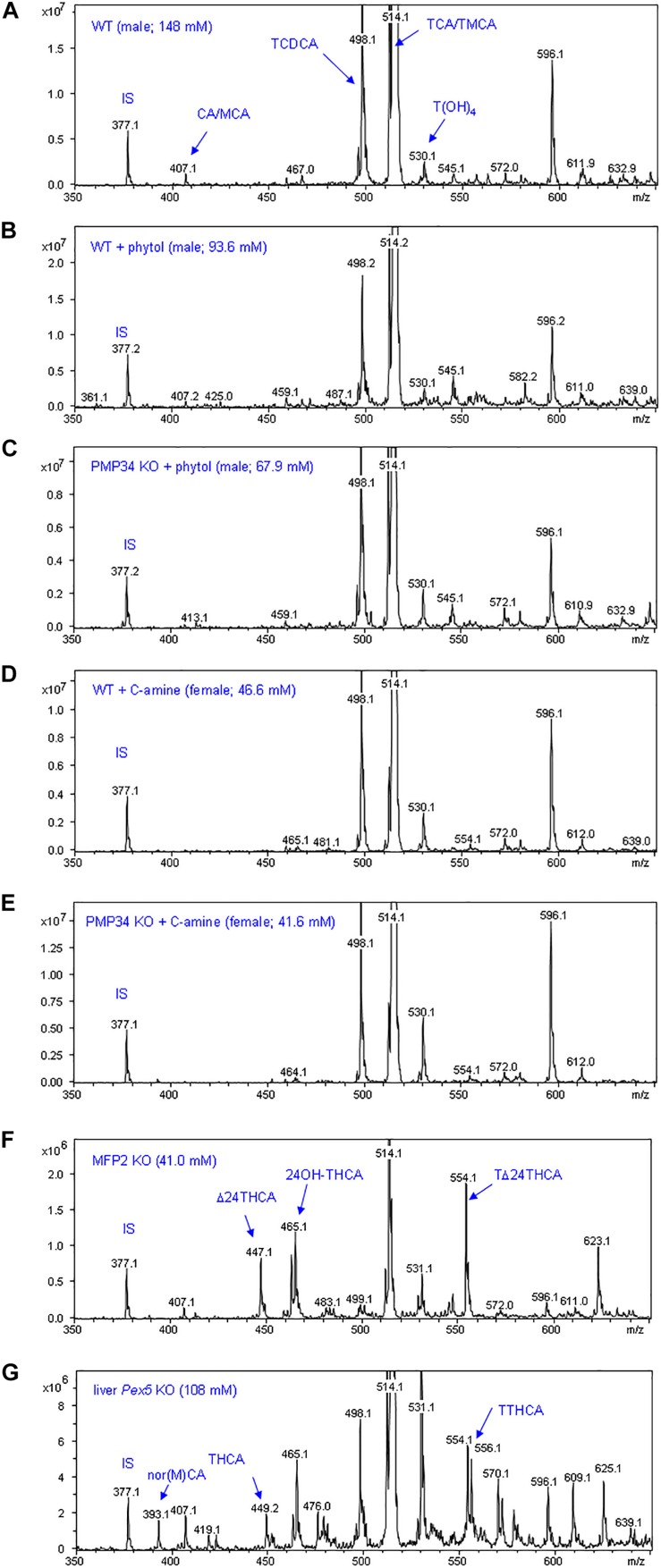
Biliary bile acid profiles of PMP34 KO mice. Panels **(A–E)** show MS-scans [350-650 m/z; negative mode; smoothed (0.2; 1; GA)] of bile obtained from wild type or PMP34 KO mice fed a normal or a phytol enriched diet or treated with cholesterylamine (C-amine) (gender and concentration of biliary bile acids of the individual animals is indicated in parentheses). Murine bile contains mainly tauro-conjugates, the major ones being tauromuricholic acids (TMCA, a,p-isomers) and taurocholic acid (TCA), both same m/z 514 (signal topped in all panels), followed by taurochenodeoxycholic acid (TCDCA, m/z 498) and a taurotetrahydroxycholanic acid (T(OH)_4_, m/z 530) (IS, 23-nor-3a,12a-dihydroxy-5p-cholanoic acid, m/z 377). The ion with m/z 596 represents a Na-acetate adduct of m/z 514; non-conjugated bile acids (cholic acid/muricholic acid, CA/MCA, m/z 407; deoxycholic acid, DCA, m/z 393) are hardly visible. In contrast, an abnormal profile is clearly revealed when analyzing bile of a mouse lacking MFP2 (panel **F**) with accumulation of C_27_ bile acid intermediates, such as A24-cholestenoic acid (A24-THCA, m/z 447) and its tauroconjugate (TA24THCA, m/z 554), and 24-hydroxycholestanoic acid (24OH-THCA, m/z 465), or a mouse lacking liver peroxisomes (panel **G**) with abnormal levels of trihydroxycholestanoic acid (THCA, m/z 449) and its tauroconjugate (TTHCA, m/z 556). Nor-CA/MCA (m/z 393), reported in deconjugated bile of SCPx-deficient mice, or their tauroconjugates (m/z 500), were not seen.

To complete the lipid analysis, bile composition and plasmalogen content of some tissues was investigated. No differences were seen in the biliary bile acid profiles, and abnormal C_27_ bile acid intermediates could not be detected in phytol treated KO mice, although this approach allowed to document abnormal bile acids in other mouse models for peroxisomal disorders (Figure 9). Plasmalogens levels were not altered by the phytol supplementation (Supplementary Table S2).

Finally, the concentration of cofactors TPP and CoA was measured in peroxisomes isolated from livers of phytol fed mice. Compared to unchallenged mice, a marked increase of CoA was observed but this was not different in wild type and PMP34 KO mice (Table 1). This increase is consistent with the phytol induced proliferation and with previous data. Indeed, the CoA concentration in hepatic peroxisomes of rats increases 3-fold when the animals are treated with clofibrate (0.32-0.92 mM; 4.0-70.2 nmol/g liver) (Van Broekhoven et al., 1981). The peroxisomal TPP pool was not affected by the phytol treatment (Table 1).

## Discussion

Using a gene trap ES cell line, mice heterozygous for PMP34/SLC25A17 were obtained, useful to study the physiological function of PMP34 in mammals. PMP34 deficient mice however did not display an obvious phenotype, not even after various treatments which could reveal an impaired peroxisomal functioning. Based on direct and indirect evidence, peroxisomal content of cofactors, including those proposed to be transported via PMP34, originally ATP (Visser et al., 2002) and more recently CoA, FAD, and NAD^+^ (Agrimi et al., 2012), appeared normal, both in fibroblasts and hepatocytes, and metabolic processes depending on these cofactors were not or only modestly affected. However, the finding that phytol administration caused hepatic lipid accumulation and inflammation in KO mice, is an obvious sign that PMP34 is sustaining import of a cofactor that plays a role in the degradation process of the phytol derived branched chain fatty acids, phytanic and/or pristanic acid, or that is required for the export of a pristanic acid degradation product e.g., propionyl-CoA or a shortened methylbranched acyl-CoA. Indeed, the atypical presence of pristanoyl-/phytanoyl-CoA (on their turn responsible for the increase in esterified pristanic/phytanic acid) might be secondary to the buildup of the CoA-esters of pristanic acid degradation intermediates. The tissue distribution of PMP34, with highest expression in kidney, followed by liver and adipose tissue in mouse, is consistent with a role in fatty acid oxidation, but not supportive for a selective role in bile acid formation, a liver specific process. In man, renal expression is however lower than in liver and highest mRNA levels were reported in testis (Agrimi et al., 2012).

Unfortunately, the identification of the *bona fide* PMP34 ligand(s) is hindered by many obstacles. These include the peculiar membrane properties of (isolated) peroxisomes (Van Veldhoven et al., 1987; Antonenkov and Hiltunen, 2006) precluding or hampering direct uptake and transport measurements in PMP34 deficient peroxisomes, the uncertainty about the real ligand of ABCD-transporters and their transport mechanism, linked or not linked to an intraperoxisomal activation (De Marcos Lousa et al., 2013; Okamoto et al., 2018), the contribution of peroxisomal acyl-CoA synthetases to overall phytanoyl/pristanoyl-CoA formation (Steinberg et al., 1999), the physiological role of peroxisomal thioesterases [such as ACOT6 acting on phytanoyl/pristanoyl-CoA (Westin et al., 2007)] and of nudix hydrolases converting acyl-CoA into 4′-(acyl)phosphopantetheine (Hunt et al., 2014; Shumar et al., 2018), the function of the pore-forming PMP22 (Rokka et al., 2009), and the extent by which cofactors can be co-imported bound to (folded) proteins, as detailed below.

Regarding indirect insight in the peroxisomal cofactor pools, ethylene glycol treatment and AGT measurements point to normal pyridoxal-phosphate levels in PMP34 KO mice. Based on the normal plasmalogen levels, the enzymes required for synthesis of ether lipids are functioning adequately, hence the peroxisomal concentration of their substrates (long chain acyl-CoA, dihydroxyacetone phosphate, long chain alcohol) seems unaffected. The comparable DHA levels suggest no major problem with transport of NADPH, cofactor for peroxisomal 2,4-dienoyl-CoA reductase (De Nys et al., 2001). Likewise, the apparent normal hepatic degradation of bile acid intermediates and of dicarboxylic acids and no demonstrable accumulation of VLCFA or bile acid intermediates, suggest that the peroxisomal content of adenine cofactors such as FAD, NAD^+^ and CoA, is also not altered in hepatocytes, or at least still high enough to allow dependent enzymes to operate at normal rates. Except for PUFA (see further), plasmalogens, bile acids and VLCFA were still normal in the phytol treated state. To get a more direct readout, we relied on expression of indicators/sensors targeted to peroxisomes. Peroxisomal NAD^+^ was converted into PAR, both in wildtype and deficient fibroblasts expressing PARP-SKL, while peroxisomal ATP levels, based on the use of a peroxisome targeted luciferase reporter, seem normal, at least in non-challenged fibroblasts. The first finding does not support the PMP34 mediated import of NAD^+^, as proposed by Agrimi et al. (2012); the latter finding agrees with their work, showing that PMP34 is not an ATP-transporter. Given the very low expression of PMP34 in skin, one can question, however, whether fibroblasts are an appropriate cell type for such studies. Direct determination of the CoA and TPP content of isolated liver peroxisomes did not reveal differences between normal and deficient mice, even not after a phytol-challenge. These pools, however, represent (mainly) enzyme bound-cofactors (Van Veldhoven and Mannaerts, 1986; Fraccascia et al., 2011), that might have been co-imported with these enzymes.

**FIGURE 10:**
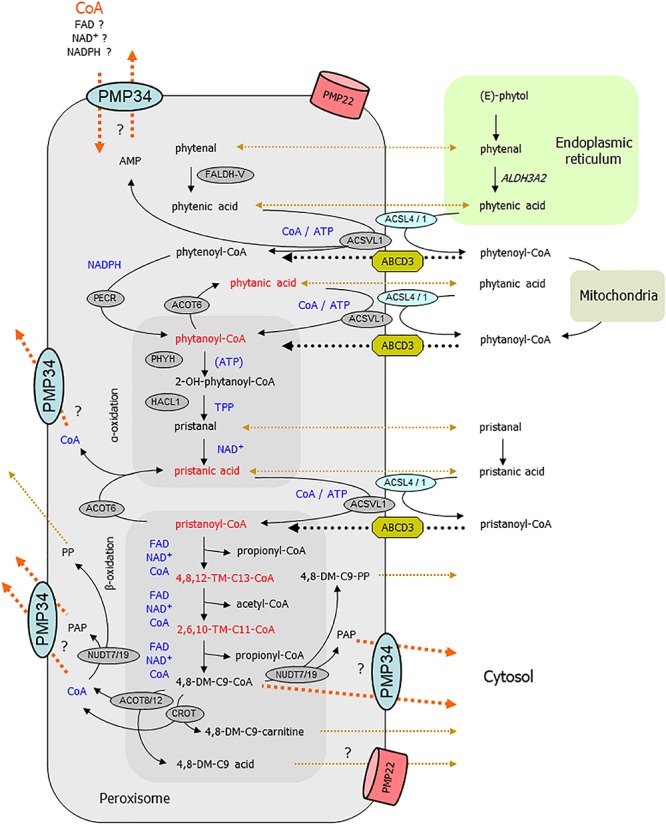
Link between PMP34 and peroxisomal phytol degradation. Schematically shown are the different steps involved in phytol breakdown and their (presumed) subcellular sites. Cofactors involved in intraperoxisomal reactions are highlighted in blue; enzymatic steps are drawn with solid black arrows; transport processes are shown with dotted lines, black for known or accepted ones, ochre-brown for non-characterized or putative ones, orange for (hypothesized) PMP34 related ones. The areas depicting reactions related to α- and β-oxidation are tinted darker. Compounds (or their hydrolysis product) depicted in red accumulate in phytol treated PMP34 knockout mice. Likely, different phytol derivatives can enter peroxisomes (phytol, 2-phytenal, 2-phytenic acid, phytanic acid, 2-phytenoyl-CoA, phytanoyl-CoA, phytanic acid), the CoA-esters presumably via ABCD3, but whether or not hydrolysis occurs during this transport is not specified. Activation of phytanic acid by ACSVL1 requires intraperoxisomal ATP/CoA (and export of generated AMP, perhaps via PMP34), but phytanic acid could also be activated at the cytosolic side of the peroxisomal membrane by ACSL4/1. In the α-oxidation process, ATP stimulates PHYH but is not required for its activity. Before pristanic acid, produced by α-oxidation, can enter the β-oxidation spiral, it needs to be activated to its CoA-ester, a CoA/ATP-dependent step which could be carried out in the peroxisomal lumen by ACSVL1 (AMP export not depicted) or in the cytosol by ACSL4/1, followed by transport via ABCD3. Subsequently, pristanoyl-CoA is degraded by three β-oxidation cycles (individual reactions are not shown) whereby the produced 4,8-dimethylnonanoyl-CoA (4,8-DM-C9-CoA) is presumed to be exported either as carnitine ester or acid, both reactions producing CoA. Similarly, the other β-oxidation products acetyl- and propionyl-CoA can be converted to carnitine esters (by CRAT) or hydrolyzed (by ACOT8/12) and result in CoA formation (not shown). CoA is not formed when 4,8-dimethylnonanoyl-CoA is cleaved by nudix hydrolases (NUDT), instead adenosine-3′,5′-diphosphate (PAP) and 4,8-dimethylnonanoyl-phosphopantetheine (4,8-DM-C9-PP) are generated. These NUDT enzymes can also act on CoA, producing phosphopantetheine (PP) and PAP. These products (MW 358, respectively 423 Da), being negatively charged, might not leak out via PMP22, but could rely on PMP34. Given the presence of shortened branched acyl-CoAs, activation of phytanic/pristanic acid is not a major issue if PMP34 is absent. Inadequate import of CoA (orange arrow), required for the SCPx reactions of the β-oxidation spiral, can explain the build-up of the upstream intermediates. Inability to export CoA, formed by the above mentioned ACOT/CROT/CRAT reactions, could reverse these reactions and slow down β-oxidation. If the CROT/ACOT8 reactions are to sluggish to handle an exces of 4,8-dimethyl-C9-CoA, PMP34-mediated export of this CoA-ester would offer another explanation for the observed metabolic phenotype. Inability to remove PAP could impact phytol degradation as well, if this compound is inhibitory to some oxidation enzymes. DM-, dimethyl-; TM-, trimethyl-; ER, endoplasmic reticulum.

Regarding the abnormal phytol breakdown, a scheme of the different metabolic steps that can take place in peroxisomes is displayed in Figure 10. The first intermediary CoA-ester derived from (*E*)-phytol that might be peroxisomal is 2-phytenoyl-CoA. As we did not detect 2-phytenic acid^9^ in liver extracts of the phytol fed KO mice, we assume that its activation (in the ER or at the cytosolic side of the peroxisomal membrane) proceeds normal, as well as its reduction to phytanoyl-CoA. If this reduction step is only catalyzed by the peroxisomal reductase PECR (Gloerich et al., 2006), this would imply that the peroxisomal NADPH content is normal in the PMP34 KO mice. In case 2-phytenoyl-CoA is reduced in mitochondria or by ER, peroxisomes would be supplied with phytanoyl-CoA or phytanic acid. If the latter is activated inside peroxisomes via very long chain acyl-CoA synthetase (ACSVL1) (Steinberg et al., 1999), a lack of peroxisomal CoA or/and ATP will cause phytanic acid accumulation. It should be noted that the peroxisomal CoA pool is not accessible to the activating enzyme(s) (Mannaerts et al., 1982; Van Veldhoven and Mannaerts, 1986).

A subsequent step, hydroxylation of phytanoyl-CoA by phytanoyl-CoA hydroxylase (PHYH), might function suboptimally due to ATP shortage. However, ATP is not an essential cofactor for PHYH, but rather a stimulatory one, and this effect has only been demonstrated *in vitro* (Croes et al., 2000). Furthermore, (accumulating) pristanic acid can only be formed via functional hydroxylation (by PHYH) and cleavage (by 2-hydroxyacyl-CoA lyase, HACL1)^10^ reactions (Casteels et al., 2003), indicating that these reactions are still functional. Consequently, peroxisomal content of the involved cofactors α-ketoglutarate/iron/ascorbic acid and TPP respectively, are not affected as partially confirmed by direct TPP measurements. Further downstream in the degradation process, ATP/CoA could be required for the intra-peroxisomal activation of pristanic acid by ACSVL1 (Steinberg et al., 1999). However, a bypass is feasible, whereby pristanic acid leaves the peroxisomes, is activated by long chain acyl-CoA synthetase ACSL4 whose active center faces the cytosol (Lewin et al., 2002), and re-enters the peroxisomes as pristanoyl-CoA. Inability to remove AMP, proposed to be exported via PMP34 (Agrimi et al., 2012), would slow down the intraperoxisomal activation of phytanic/pristanic acid, but cannot explain the accumulating CoA-esters of shortened pristanic acid intermediates.

Product inhibition in the ^–/–^ status could provide an alternative explanation for the phytanic/pristanic acid accumulation. Fatty acid profiles do reveal the presence of pristanic acid breakdown products such as 4,8,12-trimethyltridecanoate and, to a lesser extent, 2,6,10-trimethylundecanoate^11^. According to literature, these branched fatty acids are produced by peroxisomal reactions (Van Veldhoven, 2010), and their CoA-esters, if accumulating intraperoxisomally, might compete with pristanoyl-CoA for ACOX2 and other β-oxidation enzymes such as MFP2 and SCPx. The detection of these truncated pristanic acid products nevertheless implies that pristanoyl-CoA is still degraded in the phytol fed KO mice, but points at the same time to a block in their breakdown. So, the problem in the KO mouse might be situated at another level, such as the exit of β-oxidation products like acetyl-, propionyl- and 4,8-dimethylnon-anoyl-CoA. The exit of these pristanic acid metabolites however, is assumed to occur in the form of carnitine esters or in non-esterified form (Van Veldhoven, 2010), and both processes are ATP-independent. Alternatively, if CoA would be retained inside peroxisomes in PMP34 deficiency, this could counteract the carnitine exchange and perhaps also the thioesterase reaction, and result in buildup of upstream metabolites. A shortage of peroxisomal carnitine, although compatible with the observed accumulation of shortened acyl-CoAs, seems unlikely: carnitine can permeate the peroxisomal membrane (at least in broken systems) (Van Veldhoven et al., 1987; Antonenkov et al., 2004; Antonenkov and Hiltunen, 2006) and reported ligands for PMP34 are large negatively charged nucleotides (Agrimi et al., 2012). Finally, throught the action of nudix hydrolases on CoA or shortened branched chain acyl-CoAs (Hunt et al., 2014), adenosine-3′,5′-diphosphate (PAP) is generated which, as shown by Agrimi et al. (2012), can be exported by PMP34. So PAP might accumulate intraperoxisomally in the KO, but this nucleotide has never been reported to affect peroxisomal β-oxidation enzymes and destruction of CoA seems a wasteful reaction.

Interestingly, some of the CoA-esters that accumulate in the KO animals were also seen, but at a lower level, in phytol-treated wild type females (Figures 8A–D). The phytol sensitivity of the latter is due to a gender-specific ∼10-fold reduced SCPx expression (Mackie et al., 2009), pointing to a compromised SCPx activity in PMP34 deficient liver and hence, to CoA as a limiting cofactor. This fits with the proposed PMP34 exchanger properties (Agrimi et al., 2012) and the drop, although small, in the degradation of bile acid intermediates in liver slices (Figure 2C) points to the same direction. One could argue that this is contradicted by the apparent normal peroxisomal CoA pool, but this pool is likely only used by thiolases (ACAA1A and ACAA1B in mice) (Mannaerts et al., 1982; Van Veldhoven and Mannaerts, 1986), which cannot act on 2-methylbranched chain substrates (Antonenkov et al., 1997). The normal biliary bile acid profile, which also depends on SCPx, however challenges this SCPx-CoA hypothesis. Here we recall that the biliary bile acid composition was near normal in SCPx KO mouse (slightly reduced C24-bile acid levels, no C27-bile acids and a small amount of C23-bile acids and a C26-bile alcohol [3a,7a,12a-trihydroxy-27-nor-5b-cholestane-24-one)] (Kannenberg et al., 1999), a finding attributed to an alternative peroxisome independent cholesterol breakdown pathway (via 25-hydroxylation). Hence, phytol-derived pristanic acid breakdown seems more affected than bile acid formation by peroxisomal CoA shortage, but this might be related to differences in loading. Taking into account that in mammals, phytol degradation is stereospecific toward the (*E*)- or *trans-*isomer (Gloerich et al., 2006) and that the administered phytol (at 0.5% v/w chow) had a *trans/cis-*isomer ratio^12^ of 2, the animals consumed approximately 45 μmol of (*E*)-phytol daily. This is several fold higher than the maximum rate of bile acid formation, estimated at 2 μmol/day/animal (based on faecal bile acid content of cholesterylamine-treated animals; Supplementary Figures S2–S4).

Whether the drop in C20:4 and C22:6 PUFA in the livers of the phytol fed PMP34 KO mice, is also related to a SCPx-CoA problem is not known. This possibility cannot be excluded but seems less likely: synthesis of C22:6 by retroconversion appears normal in murine SCPx fibroblasts, likely compensated by ACAA1 (whereas in human RCDP fibroblasts, that lack peroxisomal ACAA1 thiolase, SCPx ensures a normal retroconversion) (Ferdinandusse et al., 2001); very long chain PUFA, as reported in liver of ACOX1 KO mice (Fan et al., 1996) and testis of MFP2-deficient mice (Huyghe et al., 2006), were not observed in the fatty acid profiles of the PMP34 KO mice; and the phytol-induced drop in PUFA is also apparent, but to a lesser extent, in the wild type animals. As far as we know, an effect on PUFA has not been described in the other phytol-fed mouse models. Perhaps the elevated intraperoxisomal acyl-CoA esters compete with the retroconversion steps. Alternatively, the effect is secondary to the massive accumulation of phytanic/pristanic acid (or their CoA-esters), given that methylbranched fatty acids have been reported to interfere with fatty acid desaturation (Wahle and Hare, 1982).

Finally, given the heterogeneity seen by EM in the livers of the phytol fed PMP34 KO mice, it cannot be excluded that the observed lipid abnormalities arise from hepatocytes with impaired protein import. We believe, however, that the healthy hepatocytes must be able to cope with the phytol metabolites and that peroxisome biogenesis is affected in a subset of cells by the lipid changes since similar findings were reported in other deficient mice, the ACOX1 KO (Fan et al., 1996) and in the MFP1/MFP2 double KO mice (Jia et al., 2003; Ferdinandusse et al., 2005). Based on the three affected mouse models, a common denominator for this apparent biogenesis defect appears to be a β-oxidation intermediate. Whether this applies as well to cell lines in which catalase-negative peroxisomes have been described, like Morris hepatoma (Mochizuki et al., 1971), rat FTO-2B hepatoma cells (Frederiks et al., 2007), or HT29 cells (derived from human colon carcinoma) (Lauer et al., 1999), is not known. Interestingly, also in a liver biopsy of an atypical RCDP patient, with an extreme accumulation of phytanic acid in plasma (940 μM versus 0.01-10 μM in controls), cytoplasmic catalase and absence of catalase-containing peroxisomes were noticed in all hepatocytes (Espeel et al., 1993). In addition to hepatocytes with cytosolic catalase, the EM pictures showed cells with increased number of peroxisomes and with enlarged peroxisomes, likely the result of increased PPARα activation. The accumulating phytol breakdown products, phytanic and pristanic acid, can indeed activate PPARα and possibly other transcription factors as well (Kitareewan et al., 1996; Zomer et al., 2000). Enlarged peroxisomes have been described in at least two other mouse models for peroxisomal protein deficiencies, such as the PMP70 (ABCD3) KO mouse (Jimenez-Sanchez et al., 2000) and the phytol fed AMACR KO mouse (Savolainen et al., 2004). The increased peroxisome proliferation in the phytol fed PMP34 KO mice as compared to the wild type mice on this diet, also points to a problem in α/β-oxidation.

In view of the SCPx-CoA hypothesis, how can the lack of an obvious phenotype in PMP34 KO mice under normal conditions be explained? Obviously, because standard rodent chow is practically devoid of phytanic and pristanic acids, no adverse effects of these isoprenoids are to be expected. Secondly, breakdown of more abundant substrates, such as bile acid intermediates and dicarboxylic acids that would result in a phenotype when not operative, is not (sufficiently) affected in our model. Thirdly, the presence of another cofactor transporting system acting as a back up system in the peroxisomal membrane, either another (low affinity) nucleotide transporter(s), a pore-forming protein allowing (limited) entry, or import of cofactor-enzyme complexes, cannot be excluded. Examples of the first one could be an NAD^+^- or FAD-transporter, or even a TPP-transporter. Indeed, it was shown that the mitochondrial solute carrier SLC25A19 is able to transport diphosphate deoxynucleotides *in vitro*, while functioning *in vivo* as a TPP-transporter (Lindhurst et al., 2006), while the *Arabidopsis* NAD^+^ carrier PXN can also transport ADP (Bernhardt et al., 2012). However, not much is known about such carriers in mammalian peroxisomes, and none have been found during proteomic analyses (Kikuchi et al., 2004; Wiese et al., 2007; Islinger et al., 2010; Gronemeyer et al., 2013). In this respect, mammalian peroxisomes differ from plant peroxisomes where three solute transporters have now been identified. Whether a pore-forming channel exists in the membrane of peroxisomes remained long time a matter of debate. PXMP2 (better known as PMP22), based on the altered membrane properties of hepatic peroxisomes of a KO mouse (Rokka et al., 2009), controls permeability of small solutes, up to 300 dalton, but would exclude CoA, NAD^+^, FAD, and ATP. During protein import a transient pore might be created, likely governed by PEX5 (Erdmann and Schliebs, 2005). GIM5 proteins, the presumed trypanosomal orthologs of PEX11, seem also to control peroxisomal membrane permeability and to be endowed with pore properties (Voncken et al., 2003). Biochemically, it seems however counterproductive to let metabolism depend on biogenesis. Forthly, cofactors could reach the lumen by import of cofactor-enzyme complexes, at least TPP seems to be transported complexed to HACL1 (Fraccascia et al., 2011). Finally, given the organization of peroxisomal β-oxidation, with an ABCD-dependent uptake of CoA-ester (see Figure 9), a fraction of the esters taken up can in fact be processed in the absence of a CoA-transporter if sufficient CoA is released from the substrates themselves (or from primary oxidation products formed via ACAA1).

If a low amount of adenine nucleotides can be preserved in the peroxisomes by any of the above described systems, this would suffice to keep the organelles functional under basal conditions. This idea is in agreement with the oxidation experiments in cultured cells that failed to reveal any severe defect, even when phytanic/pristanic analogs were tested. It should be noted that these tests are performed at low substrate concentrations to simulate *in vivo* conditions and that, given the position of the radioactive label, only the first cycle of β-oxidation is measured, or for 3-methyl-branched fatty acids, only the α-oxidation step. Hence, any impairment downstream of the first cycle will remain unnoticed. At least, the cellular data are consistent with the absence of an obvious phenotype in the unchallenged mice. When the system is overloaded (e.g., phytol), transport or intraperoxisomal generation of the required cofactors would become a limiting factor.

Summarizing, based on the profound phytanic and pristanic acid accumulation occuring in PMP34 deficient mice upon phytol-administration, together with presence of pristanic acid degradation intermediates (also as CoA-esters), an impaired intra-peroxisomal β-oxidation cycling, due to a decrease in the SCPx accessible CoA, is proposed as working hypothesis. Under normal conditions, by unknown mechanisms, cofactors are however sufficiently high to maintain basal oxidation rates. Given the fact that man has a much higher exposure to phytanic and pristanic acid as compared to rodents, and that phytol treatment is required to reveal serious pathology in other mouse models with peroxisomal enzyme deficiencies [e.g., AMACR (Savolainen et al., 2004), SCPx (Seedorf et al., 1998), PHYH (Ferdinandusse et al., 2008) or HACL1 (Mezzar et al., 2017)], we believe that PMP34 deficiency should be added to the list of human disorders in which phytanic acid can accumulate. So far this list contains single enzyme deficiencies like Refsum disease (PHYH deficiency), racemase, MFP2 and SCPx deficiency, as well as biogenesis disorders such as Zellweger syndrome, neonatal adrenoleukodystrophy, infantile Refsum disease and rhizomelic chondrodysplasia punctata. Likely patients harboring a PMP34 deficiency would only present with symptoms at a later age, as is the case with adult Refsum disease.

## Author’s Note

Recently, Kim et al. (2020) reported on the presence of two Slc25a17 related and apparently redundant genes in zebrafish. A real knock-out model was not created, but in morpholino-silenced embryos consumption of the yolk sac lipids and inflation of the swim bladder was impaired, with retention of lipid droplets in the latter organ. Small changes in VLCFA (10% increase C24/C22 at 3 days) and plasmalogens (15% decrease at 3.5 days) were documented. In vitro assays with both recombinant proteins showed transport of CoA and AMP in reconstituted liposomes, not of ATP, ADP or NAD+. Injection of CoA in one cell stage embryos, together with the morpholinos directed against either transporter, restored the swim bladder function partially.

## Data Availability Statement

The datasets generated for this study are available on request to the corresponding author.

## Ethics Statement

The animal study was reviewed and approved by Ethical Committee for Animal Experimentation (ECD) - KU Leuven.

## Author Contributions

EVA: mice breeding and manipulation, histology, biochemical analysis, and acquisition of data. EDS: mice breeding, oxidation experiments, and biochemical analysis. SY: generation ES cells and chimeric mice. AZ: guidance embryonal analysis. MF: design plasmids and generation of antibodies. ME: catalase cytochemistry and electronmicroscopy. MB: supervision histology and editorial advice. PVV: study concept and planning, mice breeding and dietary manipulation, generation of fibroblasts, oxidation experiments, vector design, acyl-CoA analysis, supervision biochemical work and molecular biology, and drafts.

## Conflict of Interest

The authors declare that the research was conducted in the absence of any commercial or financial relationships that could be construed as a potential conflict of interest.

## Footnotes

1 Some of the related data were described in a Ph.D. thesis by E. Van Ael (Studies of the mammalian peroxisomal membrane protein PMP34; Doctoral thesis in medical sciences, KU Leuven, 2008).

2 www.genetrap.org

3 http://baygenomics.ucsf.edu/protocols/comp1/blastocyst.html

4 Mice or cells in which both alleles are inactivated by an insertion, are referred to as knockout for the sake of simplicity.

5 The exon 1-βgeo fusion transcript cannot be detected with the Northern probe used, which covered exons 4 to 9

6 PMP34 mRNA levels in ^–/–^ pups were 0.35 ± 0.31% of those in wild type liver (*n* = 5). Whether these low values do really reflect full length mRNA is not known. If so, the gene trap appears to be very rarely spliced out in the ^–/–^ status, resulting in a correct mRNA. Such low residual mRNA levels probably escape detection in normal Northern analysis, but might be inherent to knockout models based on intron gene trapping.

7 Although the α-isoform is predominantly expressed in liver, the β-isoform was chosen being more responsive to fasting and PPARa regulation.

8 Phytanic and pristanic acid compromised 16%, respectively 61% of the fatty acids in the hepatic cholesterylesters of a female mouse; 37% and 28%. in the higher R_*f*_ triacylglycerols.

9 In general, impaired metabolism of long chain acyl-CoA esters is reflected in the accumulation of the respective long chain fatty acids in esterified form (triacylglycerols, phospholipids). The CoA-esters themselves also increase, but being intermediates and not endproducts, their concentration is much lower and these compounds are more difficult to measure.

10 The contribution of the ER-localized HACL2 is estimated at 10% in mouse liver (Mezzar et al., 2017).

11 Due to its chain length, its incorporation into lipids will be less and its accumulation might be underestimated.

12 Based on GC-MS analysis (S. Asselberghs and PP. Van Veldhoven, unpublished data).

## Abbreviations

ABC: ATP-binding cassette
ACOX: acyl-CoA oxidase
CPT1 β: carnitine palmitoyltransferase
CYP4A: cytochrome P450 4A hydroxylase
DAB: diaminobenzidine
DHA: docosahexaenoic acid
FA: fatty acid
KO: knockout
MCFA: medium chain FA
MEF: mouse embryonic fibroblasts
MFP: multifunctional protein
PAP: adenosine-3′,5′-diphosphate
PAR: poly-ADP ribose
PARP: poly-ADP ribose polymerase
PMP: peroxisomal membrane protein
PTS: peroxisomal targeting signal
PUFA: polyunsaturated FA
RCDP: rhizomelic chondrodysplasia punctata
SCP: sterol carrier protein
THCA: trihydroxycholestanoic acid.
