# Supplementary material for: Slc25a17 Gene Trapped Mice: PMP34 Plays a Role in the Peroxisomal Degradation of Phytanic and Pristanic Acid
Source: Front Cell Dev Biol. 2020 Mar 24;8:144. doi: 10.3389/fcell.2020.00144 (PMC7106852; doi:10.3389/fcell.2020.00144)
Supplement: Supplementary file 1 [file Data_Sheet_1.pdf]

Supplementary Figures S1-S8 plus legends

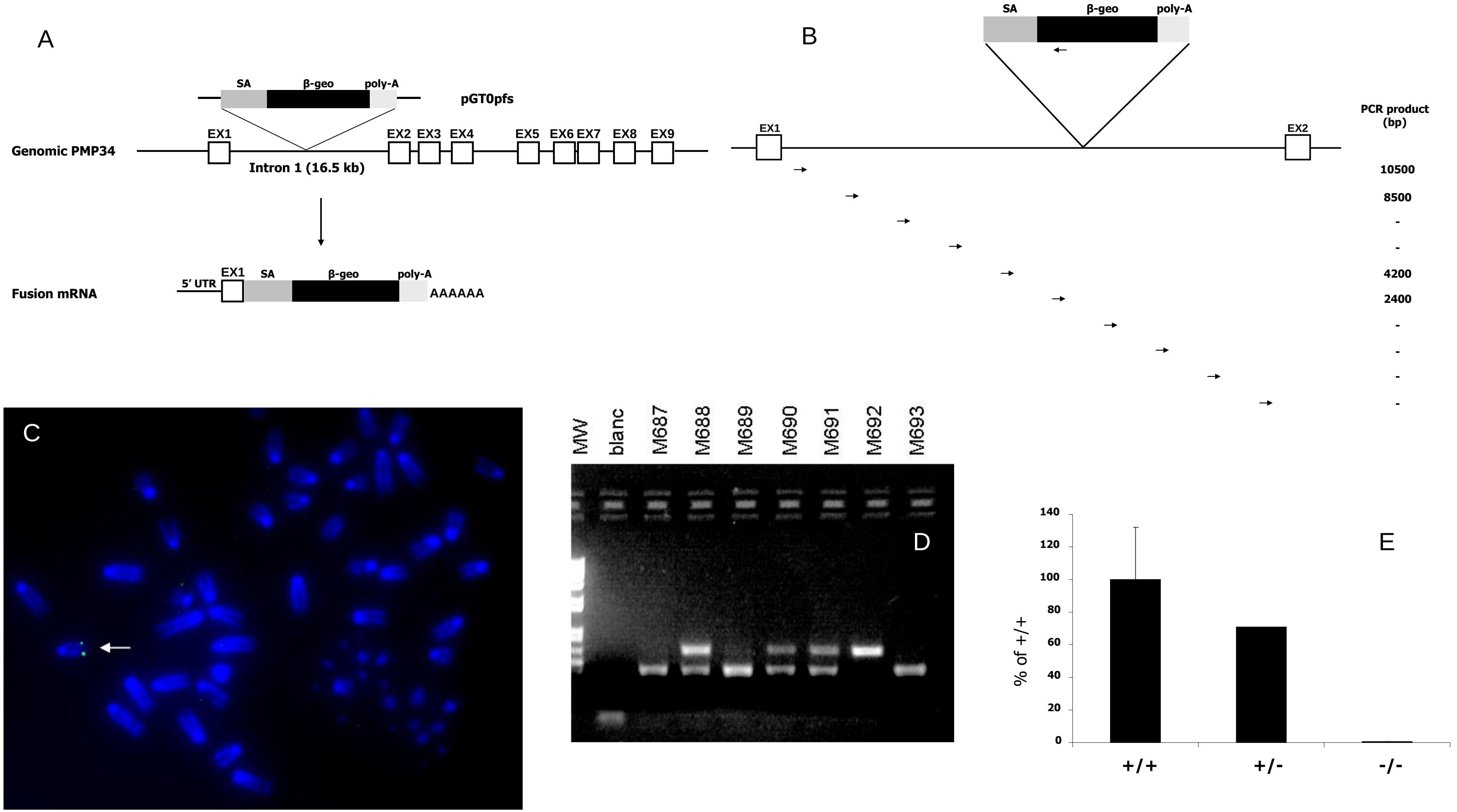

Figure S1. Targeting of the *Slc25a17* locus

(A) The chimeric *Slc25a17*$^{+/-}$ mouse was generated from ES cells in which the *Slc25a17* locus was disrupted by insertion of gene trap vector pGT0pfs into the first intron (clone XB686; BayGenomics) (exons not drawn to scale). Due to the presence of a splice acceptor site (SA) in this vector, an mRNA fusion of the first exon and β-geo, the latter coding for a fusion of β-galactosidase and neomycin phosphotransferase, is synthesized in tissues where the endogenous PMP34 promotor is active. Hence, β-galactosidase becomes a reporter for PMP34 expression. (5' UTR, 5' untranslated region; EX, exon). (B) Gene trap localization was determined by long range PCR performed on genomic DNA isolated from an ear snip of *Slc25a17*$^{+/-}$ mice using a reverse primer situated in the gene trap vector pGT0pfs (GAL-r), in combination with one of ten forward primers (Table S1), spaced approximately 1500 bp apart in intron 1 of genomic PMP34 (from left to right, primers MmPMP34-in1-s-a to j). Approximate length of PCR products resulting from the different primer combinations is indicated at the right (- indicates no product was formed). The shortest obtained long range PCR fragment (2400 bp) was cloned into the pCR-XL-TOPO vector (Invitrogen) and sequenced to determine the exact locus of integration (figure not drawn to scale). (C) FISH was performed on metaphase spreads of *Slc25a17*$^{+/-}$ MEF using a biotin-UTP labeled *Eco* RI fragment of the pGT1.8 IRESβGeo(S) vector, containing the β-geo sequence, as a probe. Representative picture of probe hybridisation (green signal) at chromosome 15 (white arrow) at the expected distance from the centromer and at a single chromosome. (D) Representative picture of a genotyping reaction of a nest of litters using duplex PCR; upper band, 667 bp amplicon derived from β-geo; lower band, 413 bp, derived from intron 1 and overlapping the pGTOpfs insertion site; pup M692 represents a knockout (MW, DNA ladder). (E) PMP34 mRNA expression in liver isolated from age-matched males $^{+/+}$ (n=5), $^{+/-}$ (n=1) and $^{-/-}$ (0.35 ± 0.31 %, n=5) mice. Real time PCR experiment was carried out in triplicate on all samples. Values were normalized to actin mRNA content and are expressed as mean ± SD.

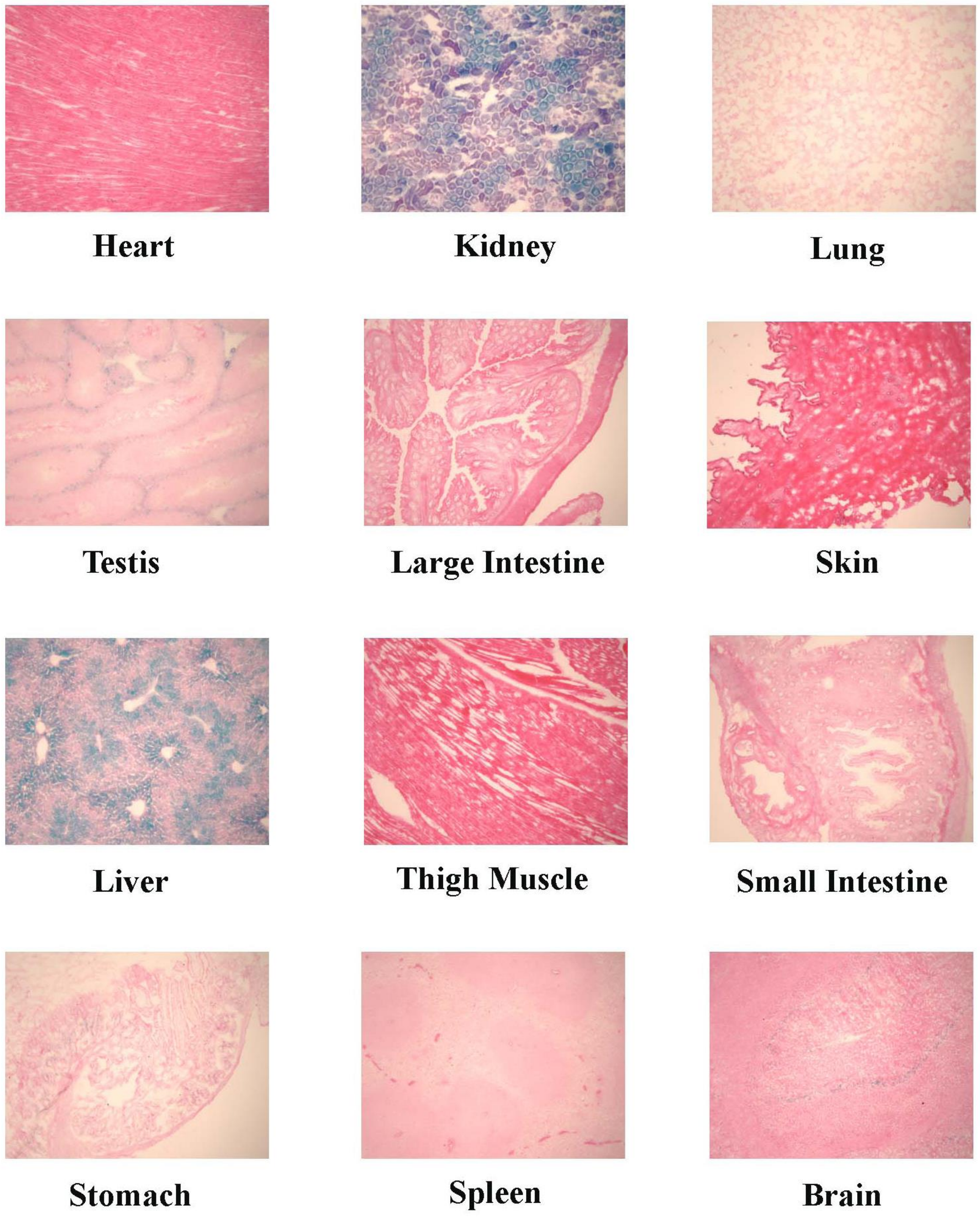

Figure S2. PMP34 expression in murine tissues

Cryosections (10 µm) from different tissues from an adult male *Slc25a17*$^{+/-}$ mouse were fixed with 2% formaldehyde/0.2% glutaraldehyde in PBS, and stained for β-galactosidase, followed by counterstaining with Nuclear Fast Red.

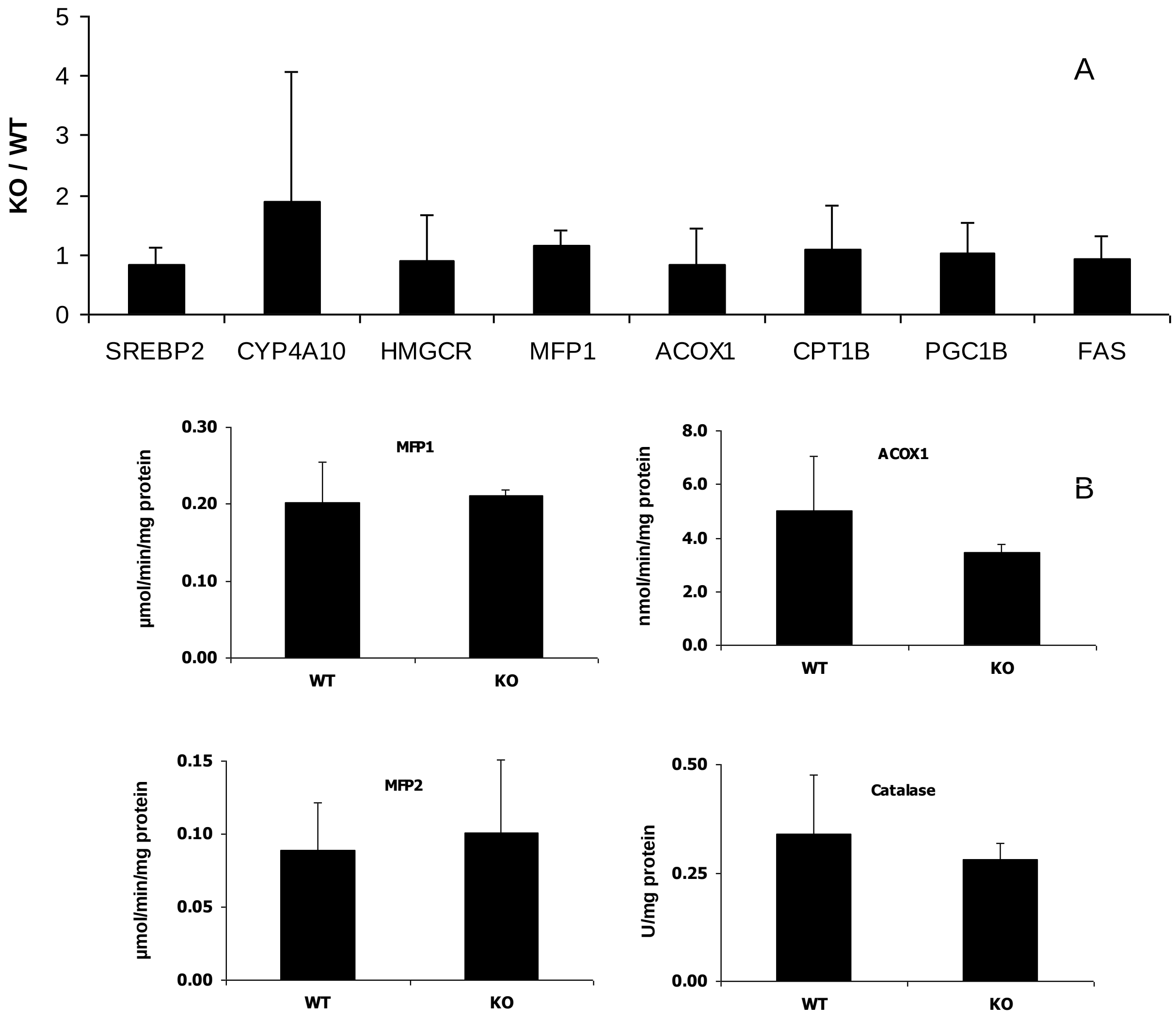

Figure S3. Evaluation of mRNA and protein levels of peroxisomal proteins and other markers in liver of PMP34 deficient mice.

Panel A. Analysis of mRNA levels in mice fed a standard diet, expressed as ratio of the level in knockout (KO) to that in wild type (WT) mice. Data shown are based on triplicate analyses of 3-4 age-matched male mice of each genotype and are expressed as mean ± SD. All expression levels were normalized to β-actin mRNA content of the samples. Abbreviations used are ACOX1, acyl-CoA oxidase 1; CPT1β, carnitine palmitoyltransferase 1β; CYP4A10, cytochrome P450, family 4, subfamily a, polypeptide 10; FAS, fatty acid synthase; HMGCR, HMG-CoA reductase; MFP, multifunctional protein; PGC1B, proliferator-activated receptor γ coactivator 1 beta; SREBP2, sterol regulatory element binding protein 2.

Panel B. Enzyme activities in liver homogenates of age-matched wild type (WT) and knockout (KO) male mice fed a standard diet. Values shown are derived from assays performed on two samples per condition and are expressed as mean ± SD.

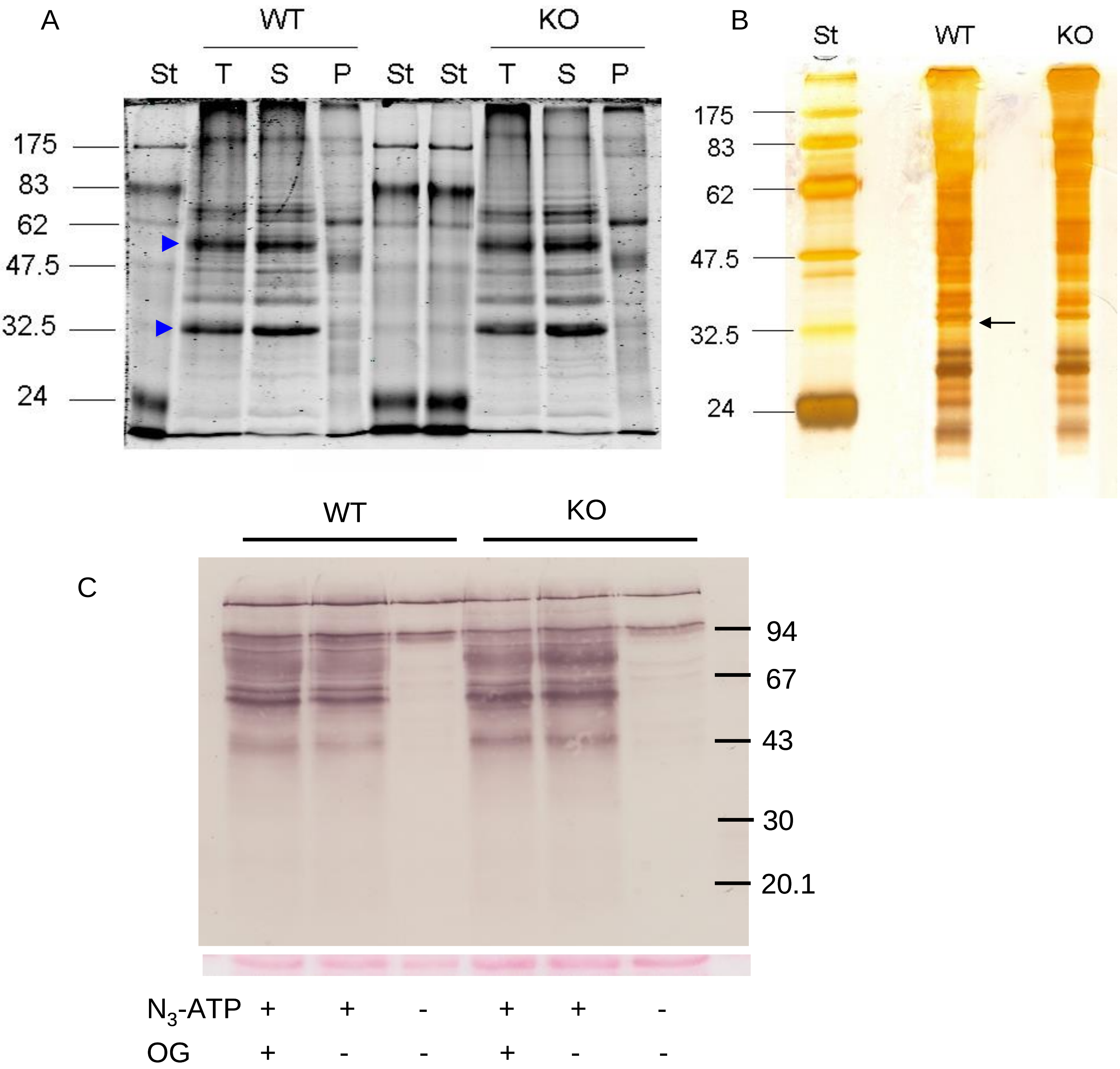

Figure S4. Protein composition of purified peroxisomes of PMP34 knockout mice

Peroxisomes were purified from pooled livers of three age matched male wild type (WT) or *Slc25a17*$^{-/-}$ (KO) mice, by Percoll and Nycodenz centrifugation, followed by isolation of the integral membrane proteins by carbonate treatment or by incubation with azido-ATP-analogues.

A. Aliquots of the carbonate diluted peroxisomes (T; 50 µg protein), and the corresponding carbonate-soluble fraction (S) and carbonate pellet (P) were analyzed by SDS-PAGE followed by Coomassie blue staining. Migration of MW standards (St) and their mass is indicated at the left; blue arrowheads indicate positions of the major proteins, catalase (60 kDa) and urate

oxidase (34 kDa). B. SDS-PAGE of the carbonate membranes (10 µg protein/lane) followed by silver staining; an extra band appears to show up in the WT-membranes (arrow). C. Streptavidin-alkaline phosphatase staining of blots prepared from purified peroxisomes (40 µg/lane) incubated with adenosine-5'-triphosphate-[γ]–4-azidoanilide-2',3'-biotin-long chain-hydrazone (N_3-ATP) under iso-osmotic conditions in presence or absence of detergent (0.2% octylglucoside, OG) and exposed to UV to evaluate the presence of ATP-binding proteins. This analogue, compared to other azido-ATP-biotin analogues tested (8-azidoadenosine-5'-triphosphate-[γ]-5(biotinamido)pentylamine, 2-azidoadenosine 5'-triphosphate-2',3'-biotin-long chain-hydrazone, and 2-azidoadenosine-2',3'-biotin-long chain-hydrazone), decorated more bands and with a higher intensity. The majority of the proteins cross-linked to N_3-ATP appeared to be matrix proteins (Meyhi E. and Van Veldhoven P.P., data not shown). Bottom panel shows the Ponceau signals associated with catalase.

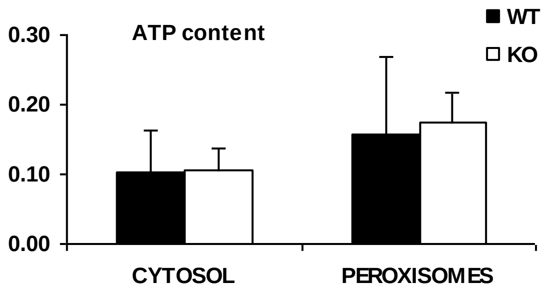

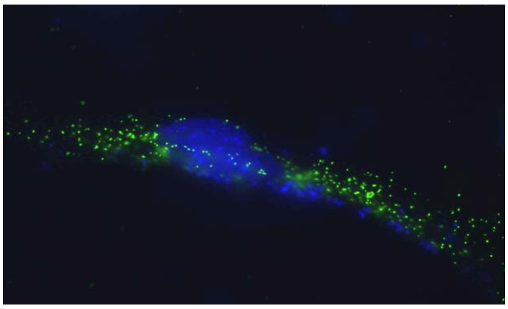

Figure S5. Evaluation of cytoplasmic and peroxisomal ATP concentration in murine fibroblasts.

Left panel. Immortalized fibroblasts derived from wild type (WT; closed bars) or PMP34 deficient (KO; white bars) embryos were cotransfected with constructs coding for β-galactosidase (pCMVβ) and firefly luciferase targeted to the cytoplasm (pGL3-CMV-luciferase) or to peroxisomes (pRSVL) in order to estimate the ATP concentration in these respective compartments. Data are derived from two independent experiments in which measurements were carried out in triplicate for each condition. Values are expressed as mean ± SD of ratios of cytoplasmic or peroxisomal luciferase activity in intact cells to luciferase activity in lysed cells of the same condition. This parameter was chosen instead of normalising the luciferase activity in intact cells for transfection efficiency by measuring β-galactosidase. Indeed, due to the endogenous galactosidase activity in the $^{-/-}$ cells, such normalisation required extra measurements and was less accurate at low transfection efficiency.

Right panel shows the immunohistostaining of wild type MEF with anti-luciferase and a FITC-conjugated secondary anti-goat antibody, 48 hours post transfection with pRSVL, and counterstained with DAPI. Transfection with pGL3-CMV-luciferase resulted in a cytoplasmic immunosignal (not shown).

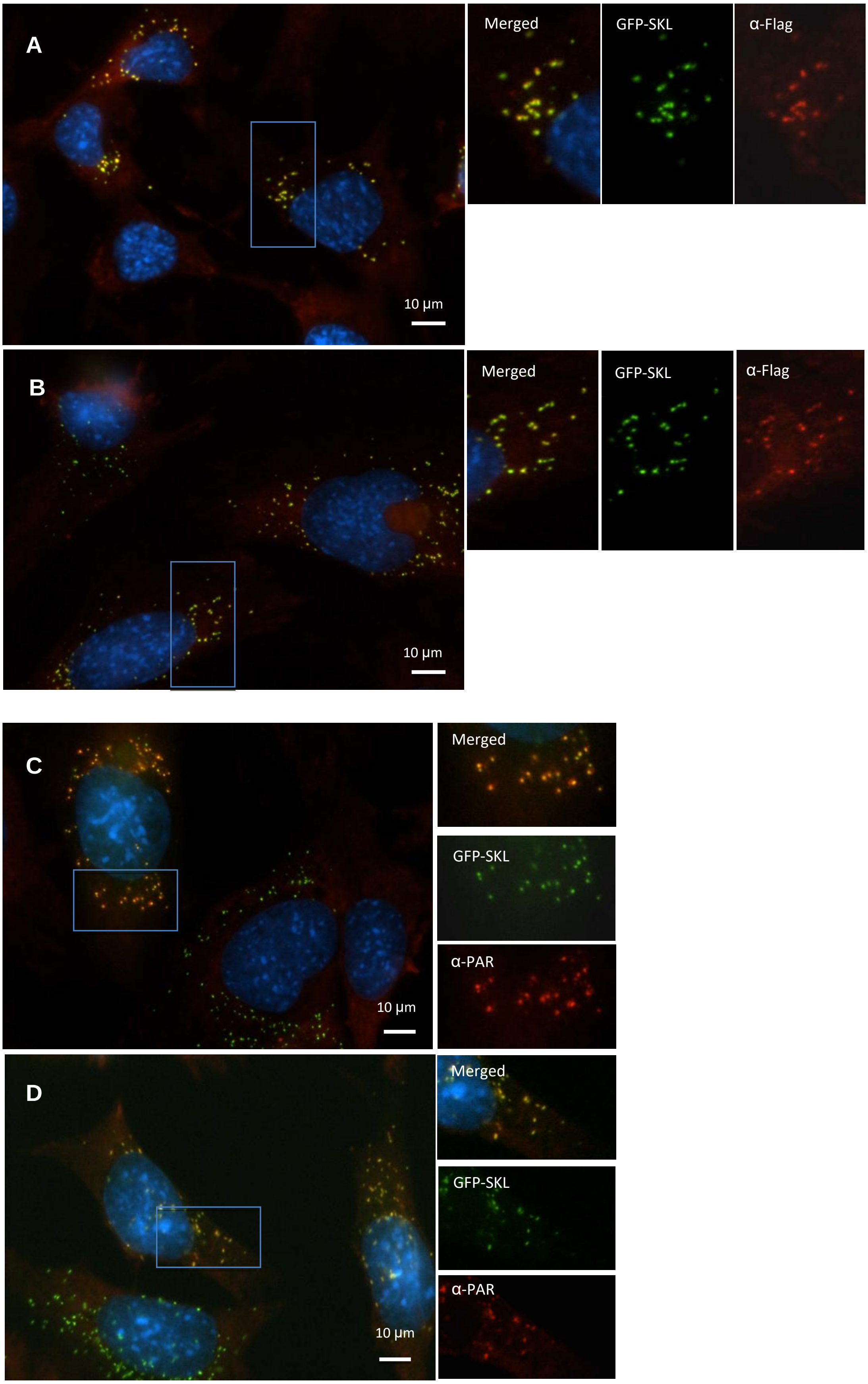
A
Merged
GFP-SKL
α-Flag
10 µm
B
Merged
GFP-SKL
α-Flag
10 µm
C
Merged
GFP-SKL
α-PAR
10 µm
D
Merged
GFP-SKL
α-PAR
10 µm

E

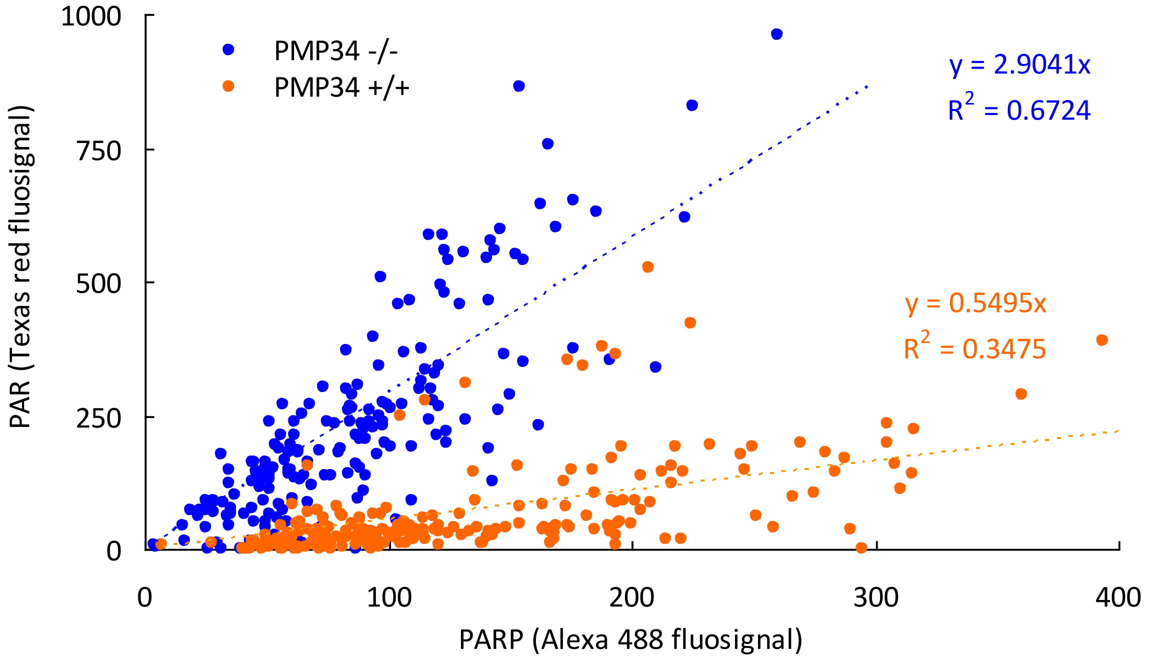

Figure S6. Evaluation of peroxisomal NAD$^+$ content in murine fibroblasts.

Immortalized wild type (A,C) or PMP34 deficient (B,D) fibroblasts were cotransfected with constructs coding for roGFP-SKL and Flag-PARP-SKL, followed by DAPI staining and immunocytochemical analysis 48 hr post transfection revealing colocalisation of GFP and PARP (A,B). Evaluation of the NAD$^+$-dependent PAR production with anti-PAR revealed a punctate pattern, overlapping with GFP, and of comparable intensity in both cell types (C,D).

To obtain more information about the relative PAR levels, wild type and PMP34 deficient MEF, transfected with the Flag-PARP-SKL encoding plasmid, were immunostained with mouse anti-PAR/anti-mouse IgG-TexasRed and rabbit anti-Flag/anti-rabbit IgG-Alexa488. Peroxisomes expressing PARP were selected with the Cell-M software (in 10-15 randomly chosen transfected cells per genotype) and signals in the green channel were recorded, followed by reading of the red channel. An intensity plot of the fluosignals associated with PARP versus PAR, whereby both genotypes were monitored under the same settings, is shown in panel E. Upon reversing the colors, by using mouse-anti-PAR/anti-mouse IgG-Alexa488 and rabbit anti-Flag/anti-rabbit IgG-TexasRed, PAR/PARP ratios were again higher in the KO cells. Based on three separate experiments, the calculated PAR/PARP slope in the KO cells was 3.57 ± 1.90 fold higher than in the wild type cells.

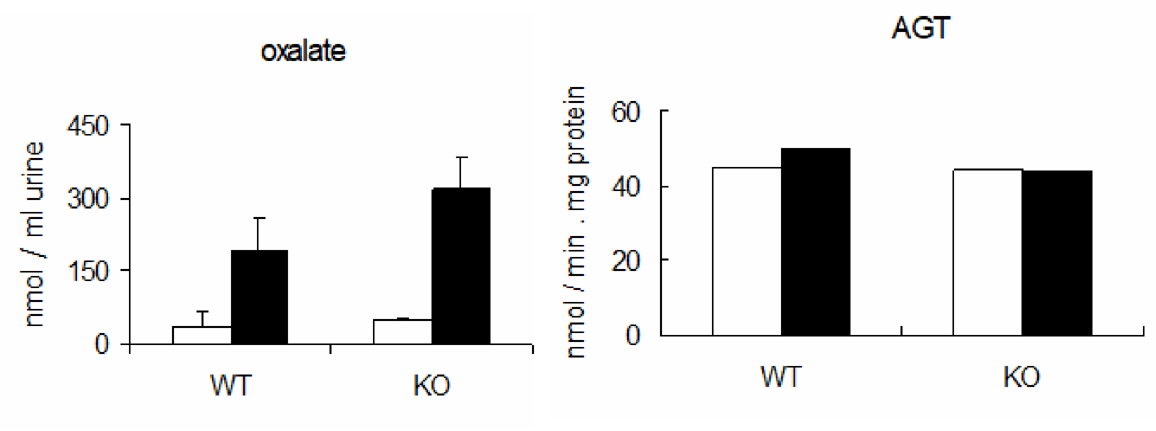

Figure S7. Analysis of pyridoxal-phosphate dependent peroxisomal processes in PMP34 deficient mice.

Left panel: Oxalate levels in urine of unchallenged (white bars) and ethylene glycol challenged (black bars) age-matched male wild type (WT) and PMP34 knockout (KO) mice. Values are based on measurements performed on two animals per condition and are expressed as mean ± SD. Right panel: Peroxisomes were purified from liver of male wild type (WT) and PMP34 knockout (KO) mice, fed a standard diet, followed by measuring AGT activity in the presence (white bars) or absence (black bars) of pyridoxal-phosphate. Values are based on single measurements per condition. Similarly, pyridoxal-phosphate did not stimulate the AGT activity in peroxisomes isolated from liver of fasted (54.9 nmol/min.mg protein) or phytol-treated (22.7 nmol/min.mg protein) male KO mice (AGT activity in preparations of the WT treated littermates was 73.6, respectively 30.0 nmol/min.mg protein).

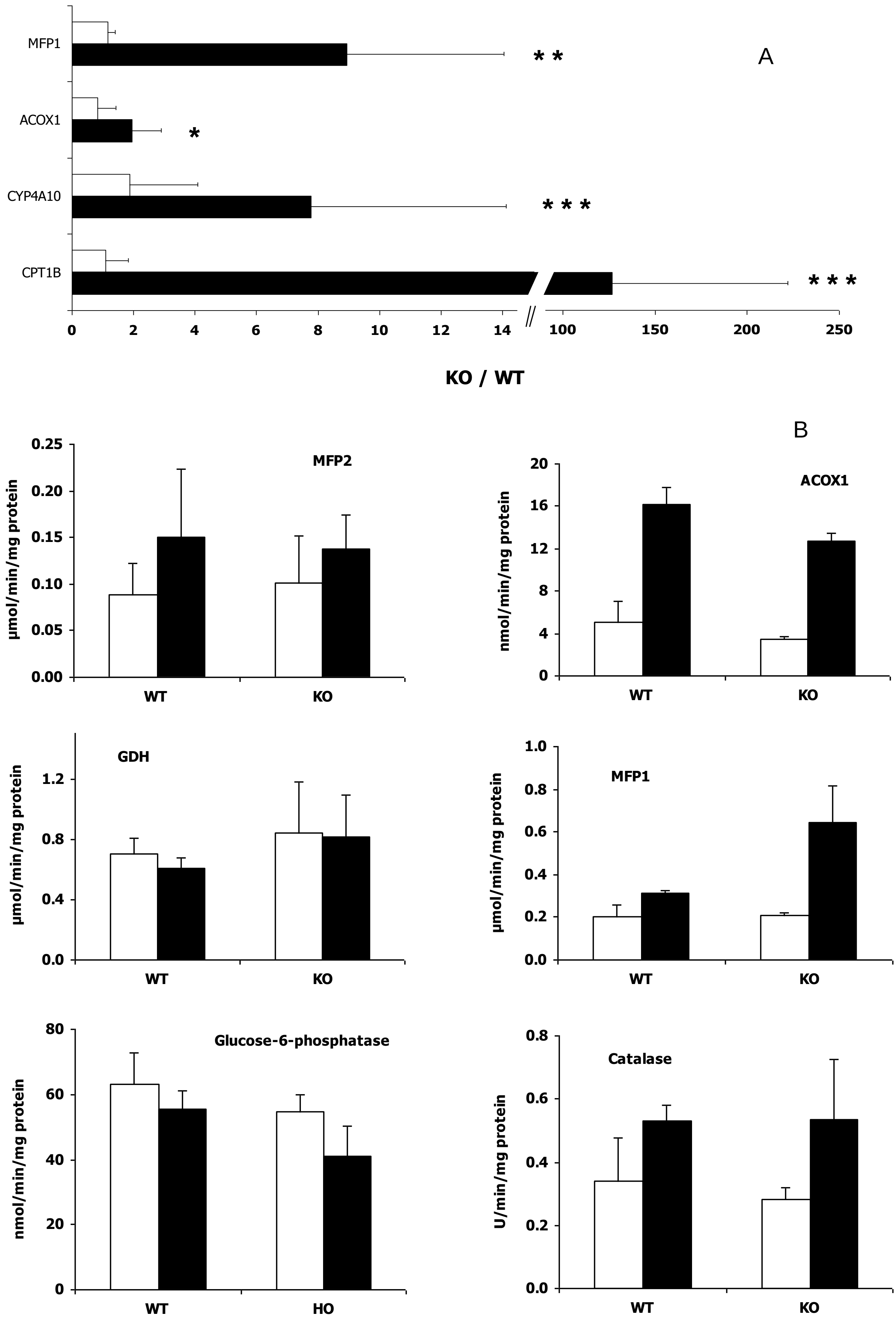
A
MFP1
ACOX1
CYP4A10
CPT1B
* *
*
* * *
* * *
0
2
4
6
8
10
12
14
100
150
200
250
KO / WT
B
MFP2
μmol/min/mg protein
WT
KO
ACOX1
nmol/min/mg protein
WT
KO
GDH
μmol/min/mg protein
WT
KO
MFP1
μmol/min/mg protein
WT
KO
Glucose-6-phosphatase
nmol/min/mg protein
WT
HO
Catalase
U/min/mg protein
WT
KO

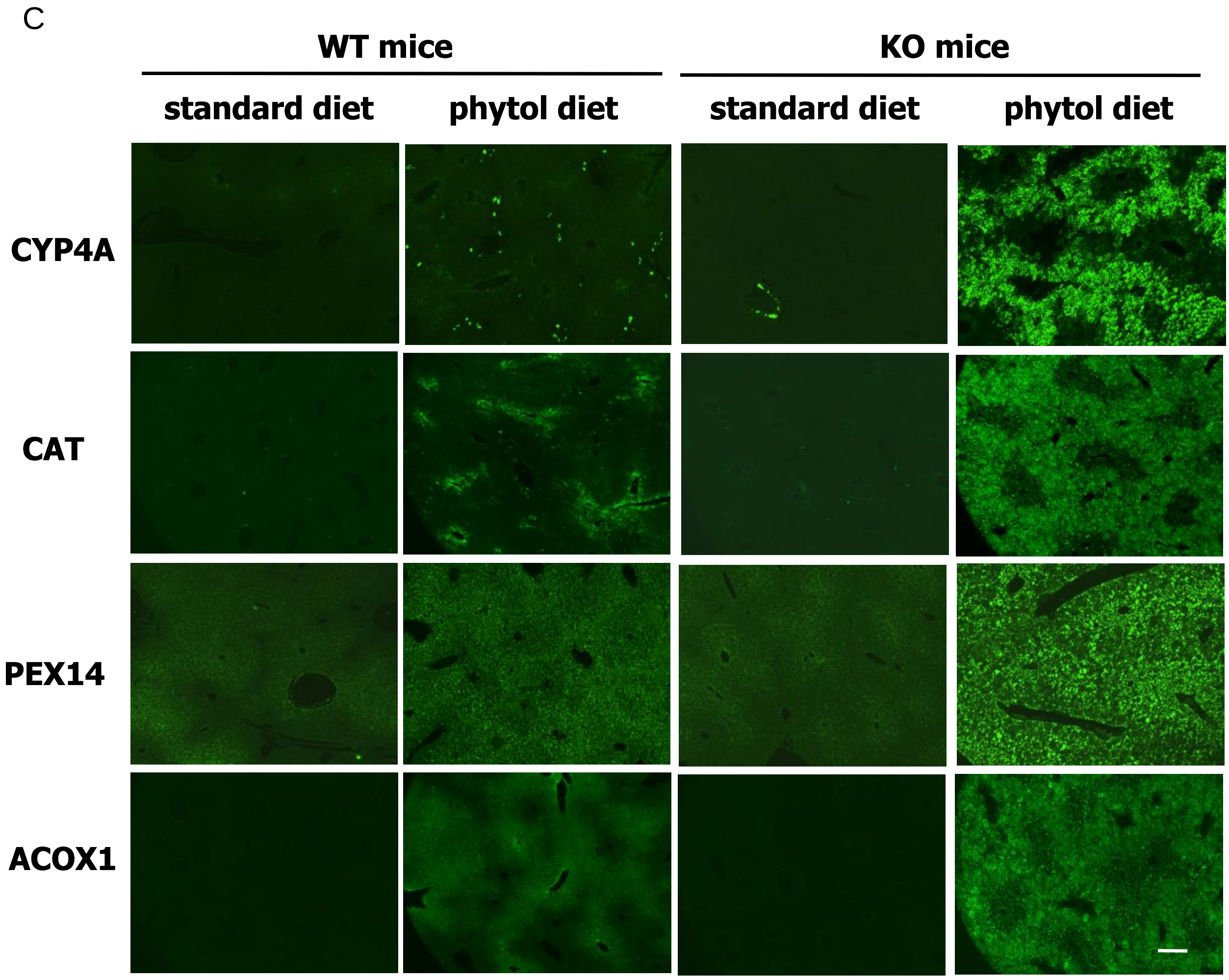

Figure S8. Effect of phytol feeding on peroxisomal enzymes and other markers in PMP34 knock out mice.

A. Hepatic mRNA expression profiling in phytol challenged PMP34 knockout mice. Levels of mRNA were analysed in age matched male mice fed standard (white bars) or phytol diet (black bars), followed by normalization to β-actin mRNA content of the samples and expressed as ratios of knockout (KO) to wild type (WT) values. Data shown are based on triplicate analyses of 4 age-matched mice of each genotype on standard diet and 3 age-matched mice of each genotype on phytol diet, and represented as mean ± SD. Data were statistically analysed using Two-Way ANOVA; ***P<0.001, **P<0.01, *P<0.1 for WT versus KO on phytol diet.

B. Enzyme activities in liver homogenates from age-matched male control (WT) and PMP34 knockout mice (KO) fed standard (white bars) or phytol enriched diet (black bars). Values are based on 2 animals per condition and are expressed as mean ± SD (values for the standard condition taken from Figure S3 to facilitate comparison between control and phytol fed condition).

C. Immunohistochemical staining of liver sections of male WT and KO mice fed standard or phytol enriched diet. For each staining, exposure time was optimized for the condition of the phytol fed KO mice and kept constant during photographing of the other conditions. As a consequence, depending on the degree of induction, signals are less visible in the standard diet conditions (scale bar 200 µm). Similar staining differences were seen in another set of phytol treated male mice, or when female mice were used.
