# Supplementary material for: Slc25a17 Gene Trapped Mice: PMP34 Plays a Role in the Peroxisomal Degradation of Phytanic and Pristanic Acid
Source: Front Cell Dev Biol. 2020 Mar 24;8:144. doi: 10.3389/fcell.2020.00144 (PMC7106852; doi:10.3389/fcell.2020.00144)
Supplement: Supplementary file 2 [file Data_Sheet_2.pdf]

Supplement 1

Prenatal death of *Slc25a17*$^{-/-}$ mice in C57BL/6 background

During the initial intercrosses of heterozygous mice on a mixed genetic background (129/OlaHsd and C57BL/6) no knockout pups were born, also not after further inbreeding in the C57BL/6 background. Examination of the prenatal stages in the C57BL/6 background failed to reveal the presence of KO embryos until $E_{14.5}$, when one knockout was found (n=30, significant departure from Mendelian ratios, χ^2 P<0.025), smaller and paler than the $^{+/+}$ or $^{+/-}$ embryos isolated during the same dissection (Figure S1-1A). MEF were prepared from these embryos and real time PCR analysis revealed a 50% reduction of PMP34 mRNA levels in heterozygous MEF and absence (0.05 ± 0.02%) of PMP34 mRNA expression in knockout MEF (Figure S1-1B). The morphology of major organelles in $^{-/-}$ MEF, based on staining for markers for peroxisomes (anti-PEX14), nuclei (anti-nucleoporin p62), Golgi apparatus (anti-GM31), mitochondria (Mitotracker), and ER (anti BIP/GRP) looked normal and similar to that seen in $^{+/+}$ MEF (Figure S1-2). No defect in the oxidation of very long chain fatty acids, either saturated (C24:0) or polyunsatured (C24:6)), 2-methyl- or 3-methylbranched fatty acids, or 2-hydroxy-fatty acids could be observed in these primary MEF (Figure 2A).

The knockout's survival to $E_{14.5}$ was considered an exception, given the abnormal Mendelian ratio. Indeed, embryonic death remained evident at $E_{13.5}$ (0 KO embryos found, n=13, χ^2 P<0.1) and at $E_{12.5}$ (0 KO embryos found, n=31, χ^2 P<0.01).

In order to increase the chances of finding knockout embryos, backcrossing to a Swiss Webster genetic background was commenced at this point, as litters from this strain are almost twice as large as those from mice of the C57BL/6 background. From the 2nd until the 5th generation, intra-uterine degeneration of the knockout embryos was evident at $E_{7.5}$ (11 knockout embryos found, n=139, significant departure from Mendelian ratios, χ^2 P<0.001) and $E_{6.5}$ (Figure S1-1). Only at the blastocyst stage ($E_{3.5}$), a normal Mendelian inheritance was observed (27% $^{-/-}$, 51% $^{+/-}$, 22% $^{+/+}$; n = 108). Knockout $E_{3.5}$ blastocysts placed in explant culture had no difficulties hatching from their zona pellucida and developed normally into inner cell mass and trophoblast outgrowth cells until day 5 in culture (Figure S1-4). From these data, embryonic death of the PMP34 knockout mice was estimated at $E_{4.5}$ or $E_{5.5}$.

For several reasons, the observed embryonal lethality is unlikely to be related to PMP34 deficiency. Firstly, death at such early time point in development is usually caused by mutations in genes involved in cell-cycle control, maintenance of chromosome stability, RNA/DNA binding and DNA repair/recombination [1]. Secondly, deficiencies in peroxisomal proteins reported sofar never resulted in embryonic death, even not in mouse models lacking peroxisomes altogether

[2,3], while functional peroxisomes are present in the KO fibroblasts (see above). Finally, upon further breeding in the Swiss background, we identified two *Slc25a17*$^{-/-}$ pups (5th and 8th Swiss generation) surviving into adulthood, and that gave rise to a colony without embryonic problems. Despite a large amount of work, the reason of death of the PMP34 KO mice within the C57Bl6 background remained unclear. Co-segregation of the trapped *Slc25a17* gen with a mutation in another essential gene is proposed as a possible explanation for this phenomenon. Based on probability of recombination (2 viable pups out of 203 pups, born during heterozygous intercrossings from 5th to 8th generation), the responsible locus would be located approx. 1 Mb up- or downstream from *Slc25a17*, but at first sight no clear candidates were found in these regions. Whereas gene traps sometimes do have unforeseen outcomes like exposing cryptic splice sites [4] or compensation for the trap by an alternative splicing [5,6], we are not aware of other reported cases on such anomalous lethality, but our findings might alert other investigators.

1. Stanton JA, Macgregor AB, and Green DP (2003) Gene expression in the mouse preimplantation embryo. Reproduction. 125, 457-468.
2. Baes M, Gressens P, Baumgart E, Carmeliet P, Casteels M, Fransen M, Evrard P, Fahimi D, Declercq PE, Collen D, Van Veldhoven PP, and Mannaerts GP (1997) A mouse model for Zellweger syndrome. Nat. Genet. 17, 49-57.
3. Faust PL and Hatten ME (1997) Targeted deletion of the PEX2 peroxisome assembly gene in mice provides a model for Zellweger syndrome, a human neuronal migration disorder. J. Cell Biol. 139, 1293-1305.
4. Osipovich AB, White-Grindley EK, Hicks GG, Roshon MJ, Shaffer C, Moore JH, and Ruley HE (2004) Activation of cryptic 3' splice sites within introns of cellular genes following gene entrapment. Nucleic Acids Res. 32, 2912-2924.
5. Voss AK, Thomas T, and Gruss P (1998) Compensation for a gene trap mutation in the murine microtubule-associated protein 4 locus by alternative polyadenylation and alternative splicing. Dev. Dyn. 212, 258-266.
6. Ludes-Meyers JH, Kil H, Nunez MI, Conti CJ, Parker-Thornburg J, Bedford MT, and Aldaz CM (2007) WWOX hypomorphic mice display a higher incidence of B-cell lymphomas and develop testicular atrophy. Genes Chromosomes Cancer 46, 1129-1136.

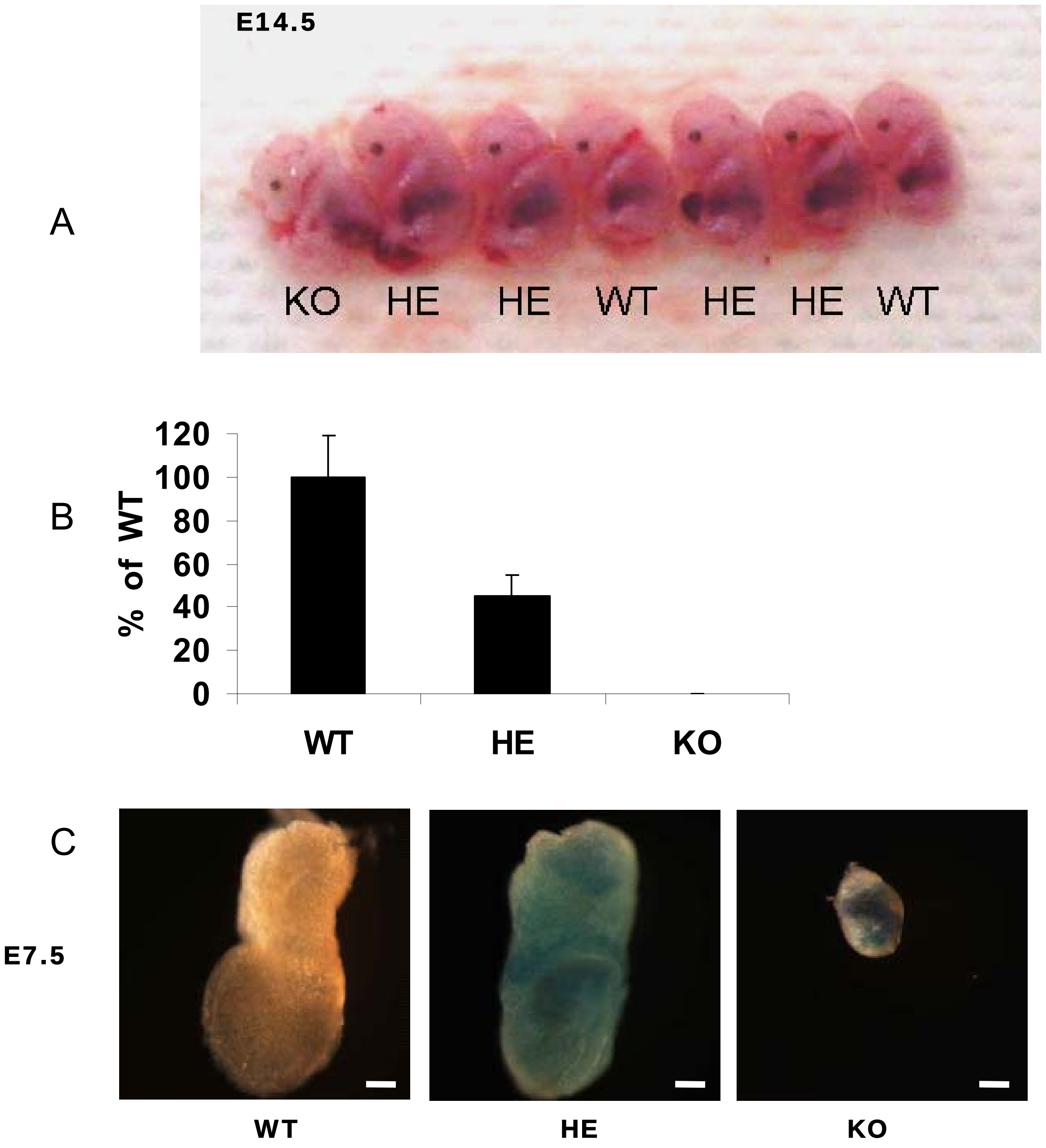

Figure S1-1. Analysis of postimplantation PMP34 knockout embryos (C57Bl/6 background).

Panel A. Picture of embryos removed at E14.5 from a pregnant female (C57BL/6 genetic background), with a single knockout (KO) embryo, paler and smaller than the heterozygous (HE) or wild type (WT) embryos. Panel B. PMP34 mRNA expression in MEF isolated from $E_{14.5}$ wild type (WT), heterozygous (HE) and knockout (KO) embryos in C57Bl/6 genetic background. Real time PCR experiments were carried out in triplicate on single samples. Values were normalized to 18S rRNA (TaqMan Ribosomal RNA Control Reagents Kit, Applied Biosystems) and expressed as mean ± SD (KO value 0.05 ± 0.02%). Panel C. β-galactosidase stained whole mount $E_{7.5}$ embryos (mixed C57BL/6 - Swiss Webster genetic background). Scale bar 100 µm.

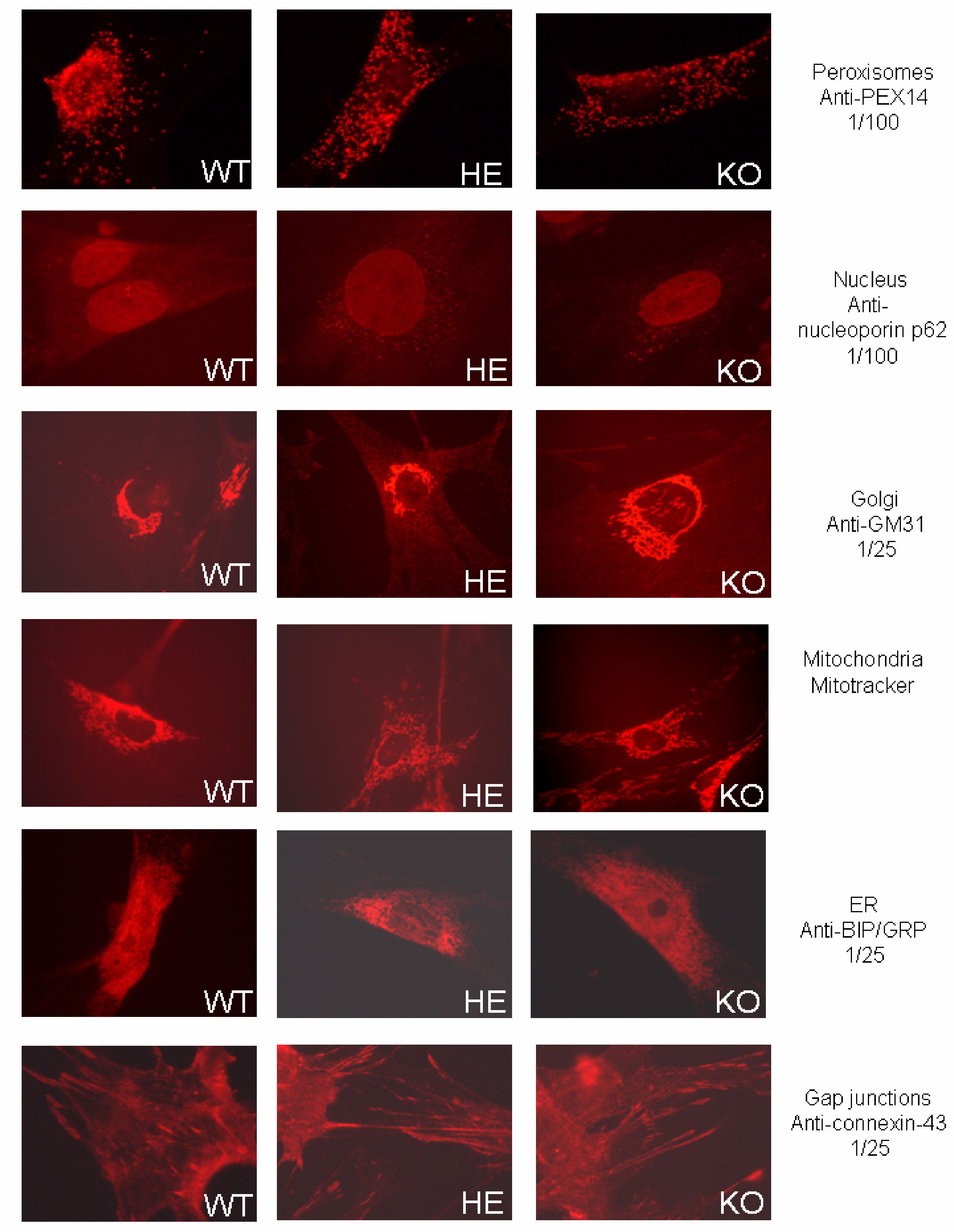

Figure S1-2. Subcellular morphology of *Slc25a17*$^{-/-}$ fibroblasts.

MEF, derived from wild type (WT), heterozygous (HE) or knockout (KO) E14.5 embryos (C57BL/6 background) were immunostained (dilution primary antiserum indicated) or treated with Mitotracker to reveal intracellular organization.

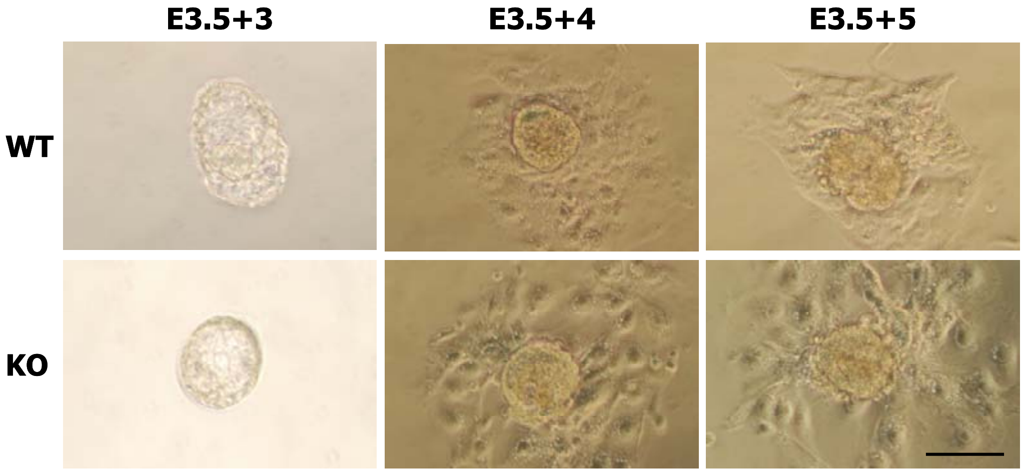

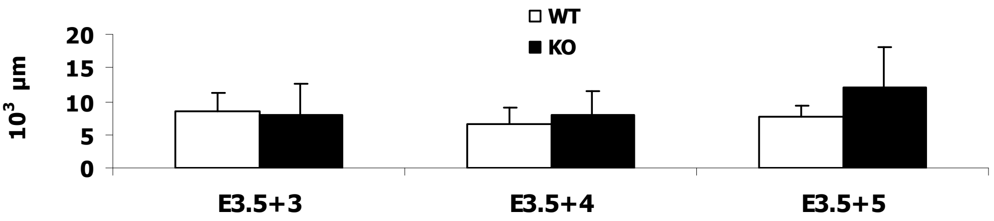

Figure S1-3. Analysis of preimplantation PMP34 knockout embryos

Upper panel: representative wild type (WT) and knockout (KO) blastocyst explant cultures isolated at $E_{3.5}$ and photographed at 3, 4 and 5 days in culture (mixed C57BL/6 - Swiss Webster genetic background). Scale bar represents 100 µm. Lower panel: mean inner cell mass area ± SD of WT (n=6) and KO (n=6) blastocyst explants measured at 3, 4 and 5 days in culture.
