# Supplementary material for: Slc25a17 Gene Trapped Mice: PMP34 Plays a Role in the Peroxisomal Degradation of Phytanic and Pristanic Acid
Source: Front Cell Dev Biol. 2020 Mar 24;8:144. doi: 10.3389/fcell.2020.00144 (PMC7106852; doi:10.3389/fcell.2020.00144)
Supplement: Supplementary file 3 [file Data_Sheet_3.pdf]

Supplement 2

Challenging of PMP34 deficient mice.

To reveal a phenotypic change in the KO mice, we attempted to increase the flux through peroxisomal pathways by dietary manipulations. In view of the guidelines and vision on animal research, only a limited number of animals per condition were used in these trials, and tests were often performed on a single gender, either male or female, depending on their availability.

PMP34 KO mice were fasted to induce fatty acid oxidation. No differences between fasted wild-type and PMP34 knockout mice were observed in terms of general appearance, weight loss (Figure S2-1), gross organ morphology or abundance of PMPs in purified liver peroxisomes (data not shown).

Clofibrate was given to induce peroxisome proliferation. As expected, upon 10 days of clofibrate administration to adult female mice (0.3% (v/w)), activities of PPARα targets increased several fold in the wild-type animals. However, the response in the KO mice was similar (Figure S2-2A). Also no differences between wild-type and KO mice were observed in general appearance, gross organ morphology or abundance of PMPs in purified liver peroxisomes (data not shown).

Like phytol (see main article), farnesol is a 3-methyl-branched isoprenoid alcohol. Although the precise metabolic fate of ingested farnesol in mammals is unknown, it is an activator of PPARα [1], possibly through metabolic intermediates that are formed in peroxisomes [2]. In agreement with others [1], farnesol administration (0.5% (w/w), 30 days) caused a slight elevation in the activities of PPARα targets; however, no differences between wild-type and KO mice were observed (Figure S2-2B). The general appearance, gross organ morphology and liver histology (haematoxylin/eosin and Oil red O stainings) of the KO and control mice were not differentially affected by farnesol (data not shown).

Different procedures were tested to increase the hepatic concentration of bile acid intermediates which are degraded by peroxisomes [2]. In agreement with others [3], we found that dietary cholesterol alone was not sufficient to increase the hepatic cholesterol content and no differences between the genotypes were observed (results not shown). Combined cholesterol and cholic acid administration (1.25% (w/w) cholesterol/0.5% (w/w) cholic acid, 21 days) increased hepatic cholesterol and cholesteryl esters, accompanied by an increase of unconjugated bile acids in bile, but wild-type and KO animals responded in the same fashion (Figure S2-3). No differences were observed between wild-type and KO mice in general appearance, gross organ morphology or on haematoxylin/eosin or Oil red O stained liver sections (data not shown). Increasing the endogenous synthesis of bile acids by feeding cholesterylamine resin, resulted in higher bile acids levels in urine, faeces and bile, but similarly in both genotypes (Figure S2-4). MS analysis of bile

did not show presence or accumulation of C_{27}-bile acid precursors in the cholesterylamine treated KO mice (see Figure 8).

Diabetis mellitus leads to an increase in dicarboxylic acids, considered to be physiological substrates for peroxisomes [2,4,5]. In mice made diabetic by streptozotocin injection (plasma glucose ≥ 4 mg/ml), no differences were observed between wild-type and PMP34 KO mice in general appearance, gross organ morphology or on haematoxylin/eosin and Oil red O stained liver sections (E. Van Ael, M. Baes and P.P. Van Veldhoven, results not shown). Another condition that results in dicarboxylic acid overload is a diet enriched in medium chain fatty acids that induces ω-hydroxylases. In mice lacking MFP1, a high fat diet based on coconut oil (enriched in lauric acid) results in weight loss, liver failure and inflammation and increased hepatic dicarboxylic acid levels [6]. Food or water uptake was similar in both genotypes, and no change in body weight was noticed in PMP34 KO mice fed a coconut oil diet (Figure S2-5A). DEXA analysis did not reveal differences in body composition and upon dissection, no particular changes in liver, kidneys or abdominal adipose tissue were seen (Figure S2-5B-D).

References

1. O'Brien ML, Rangwala SM, Henry KW, Weinberger C, Crick DC, Waechter CJ, Feller DR, and Noonan DJ (1996) Convergence of three steroid receptor pathways in the mediation of nongenotoxic hepatocarcinogenesis. Carcinogenesis 17, 185-190.
2. Van Veldhoven PP (2010) Biochemistry and genetics of inherited disorders of peroxisomal fatty acid metabolism. J. Lipid Res. 51, 2863-2895.
3. van der Veen JN, Havinga R, Bloks VW, Groen AK, and Kuipers F (2007) Cholesterol feeding strongly reduces hepatic VLDL-triglyceride production in mice lacking the liver X receptor alpha. J Lipid Res. 48, 337-347.
4. Ferdinandusse S, Denis S, van Roermund CW, Wanders RJ, and Dacremont G (2004) Identification of the peroxisomal beta-oxidation enzymes involved in the degradation of long-chain dicarboxylic acids. J. Lipid Res. 45, 1104-1111.
5. Dirkx R, Meyhi E, Asselberghs S, Reddy J, Baes M, and Van Veldhoven PP (2007) Beta-oxidation in hepatocyte cultures from mice with peroxisomal gene knockouts. Biochem. Biophys. Res. Commun. 357, 718-723.
6. Ding J, Loizides-Mangold U, Rando G, Zoete V, Michielin O, Reddy JK, Wahli W, Riezman H, and Thorens B (2013) The peroxisomal enzyme L-PBE is required to prevent the dietary toxicity of medium-chain fatty acids. Cell Rep. 5, 248-258.

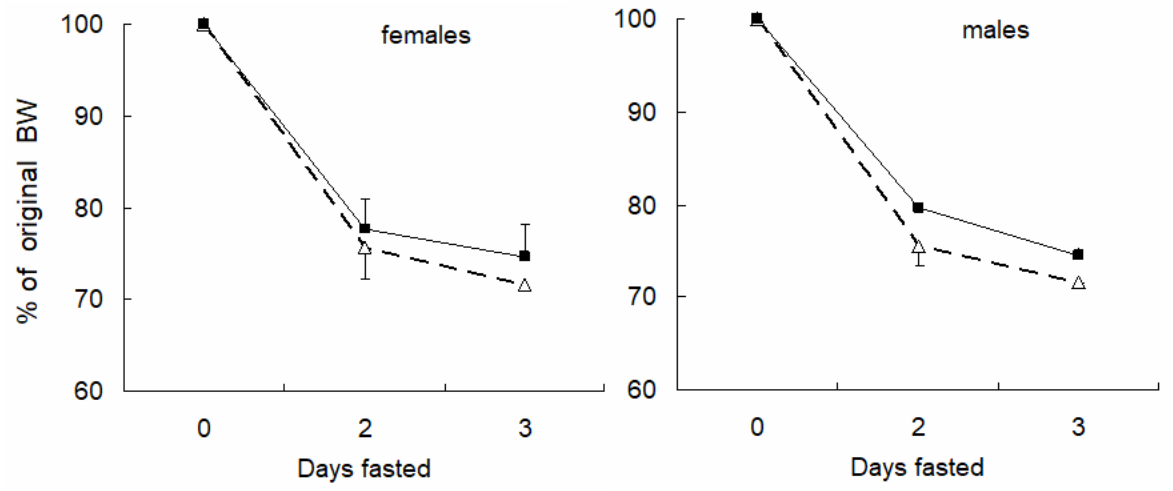

Figure S2-1. Effect of fasting on body weight in PMP34 knockout mice

Age-matched female (left panel) and male (right panel) mice, wild type (filled squares, full line) or PMP34 knockout mice (open triangles, dotted line), were fasted for 3 days and weight loss was monitored. Values, expressed as % of the initial body weight (BW), are derived from two mice per condition and are mean ± SD.

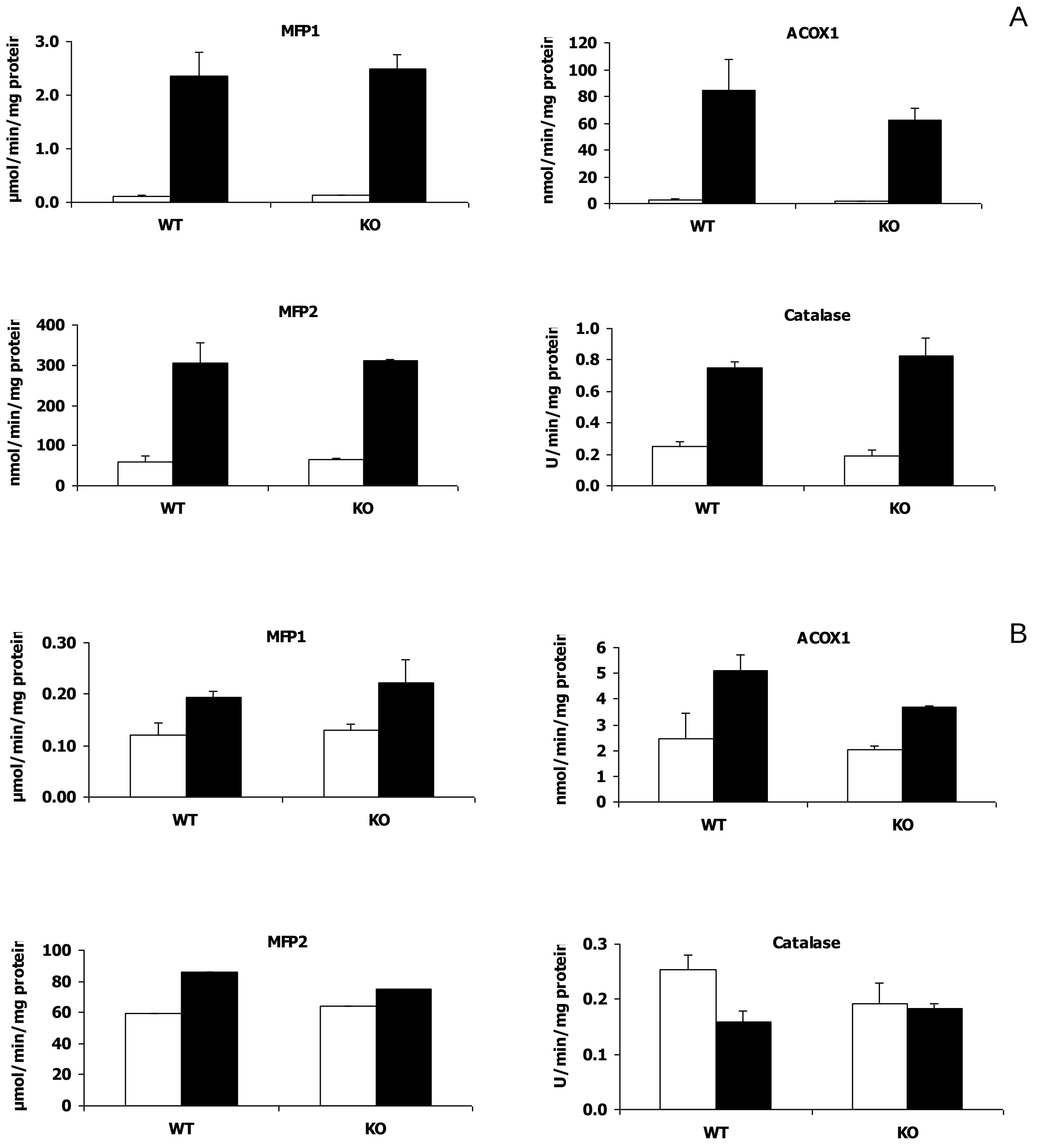

Figure S2-2. Effect of clofibrate and farnesol on peroxisomal enzymes

MFP1, MFP2, ACOX1 and catalase were measured in liver homogenates of age-matched female wild type (WT) or knockout (KO) mice. Animals were fed a standard (white bars) (A,B), a clofibrate (black bars) (panel A) or farnesol diet (black bars) (panel B). Values shown are derived from assays performed on two samples per condition and are expressed as mean ± SD.

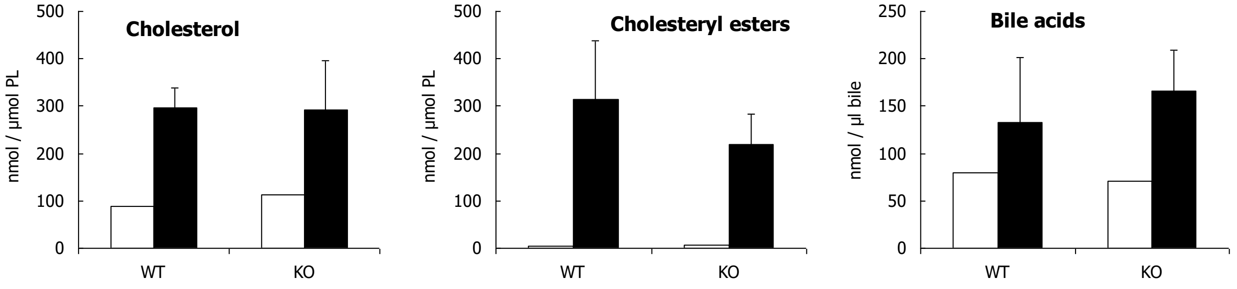

Figure S2-3. Effects of cholesterol/cholate diet on hepatic sterol content in PMP34 deficient mice.

Adult age matched male wild type (WT) and PMP34 knockout mice (KO) were fed a standard (white bars) or cholesterol/cholate enriched (black bars) diet, followed by analysis of phospholipids, cholesterol and cholesteryl esters in lipid extracts of the liver and of bile acids (non-sulfated) in bile. Values are derived from measurements performed on single samples for the standard diet, and from two samples for the cholesterol/cholate diet, in which case they are expressed as mean ± SD (PL, phospholipids).

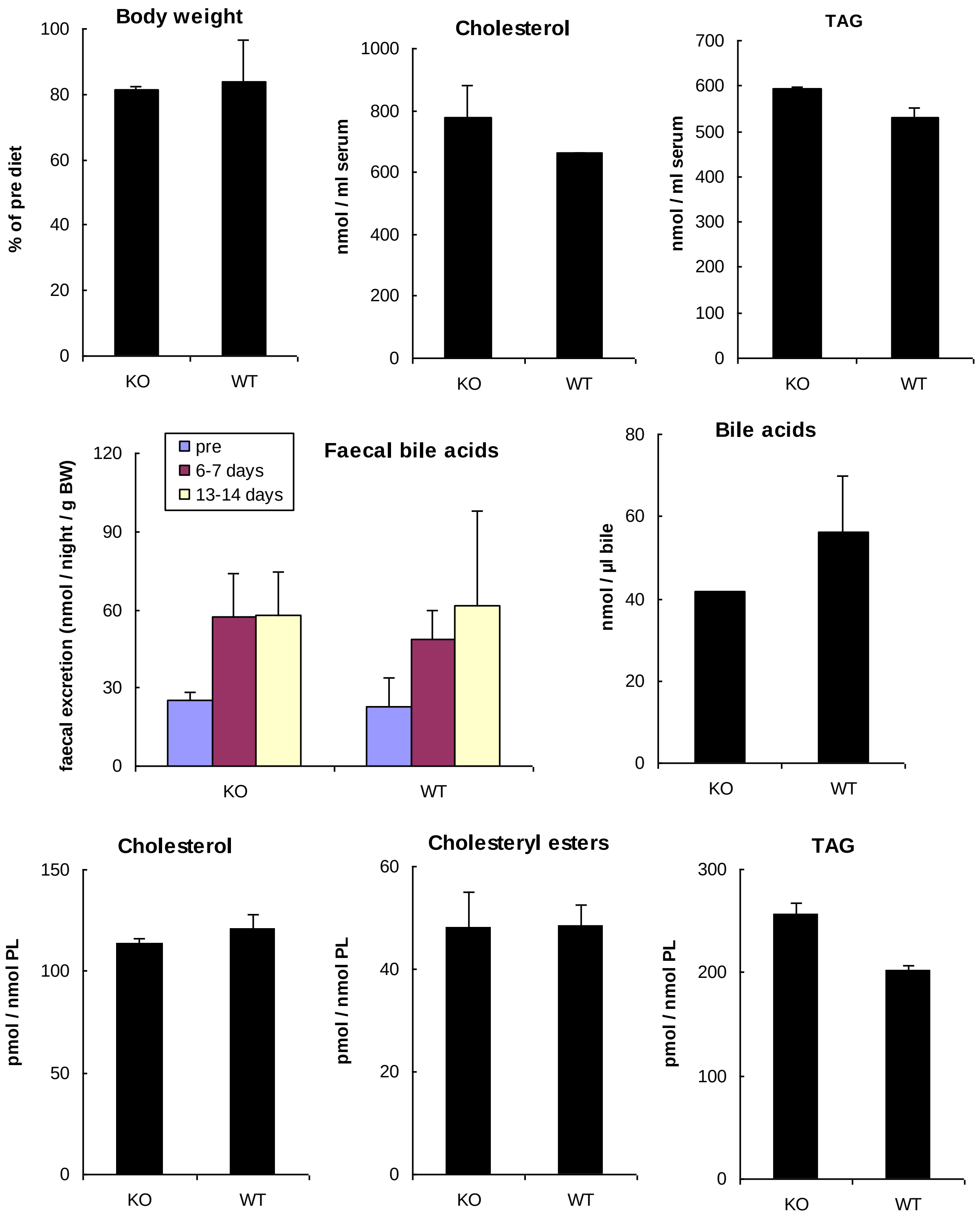

Figure S2-4. Effect of cholesterylamine on PMP34 knockout mice

Age matched knockout (KO) or wild type (WT) female mice were fed a powdered standard diet with 2% cholesterylamine during two weeks in metabolic cages. Body weights were monitored during this period, faeces were collected overnight at the start of and during the treatment; serum, bile and liver upon sacrifice at the end of the experiment. Values are derived from measurements on two animals per genotype (mean ± SD). Hepatic lipid values (bottom row) were normalized to phospholipids (PL). TAG, triacylglycerols.

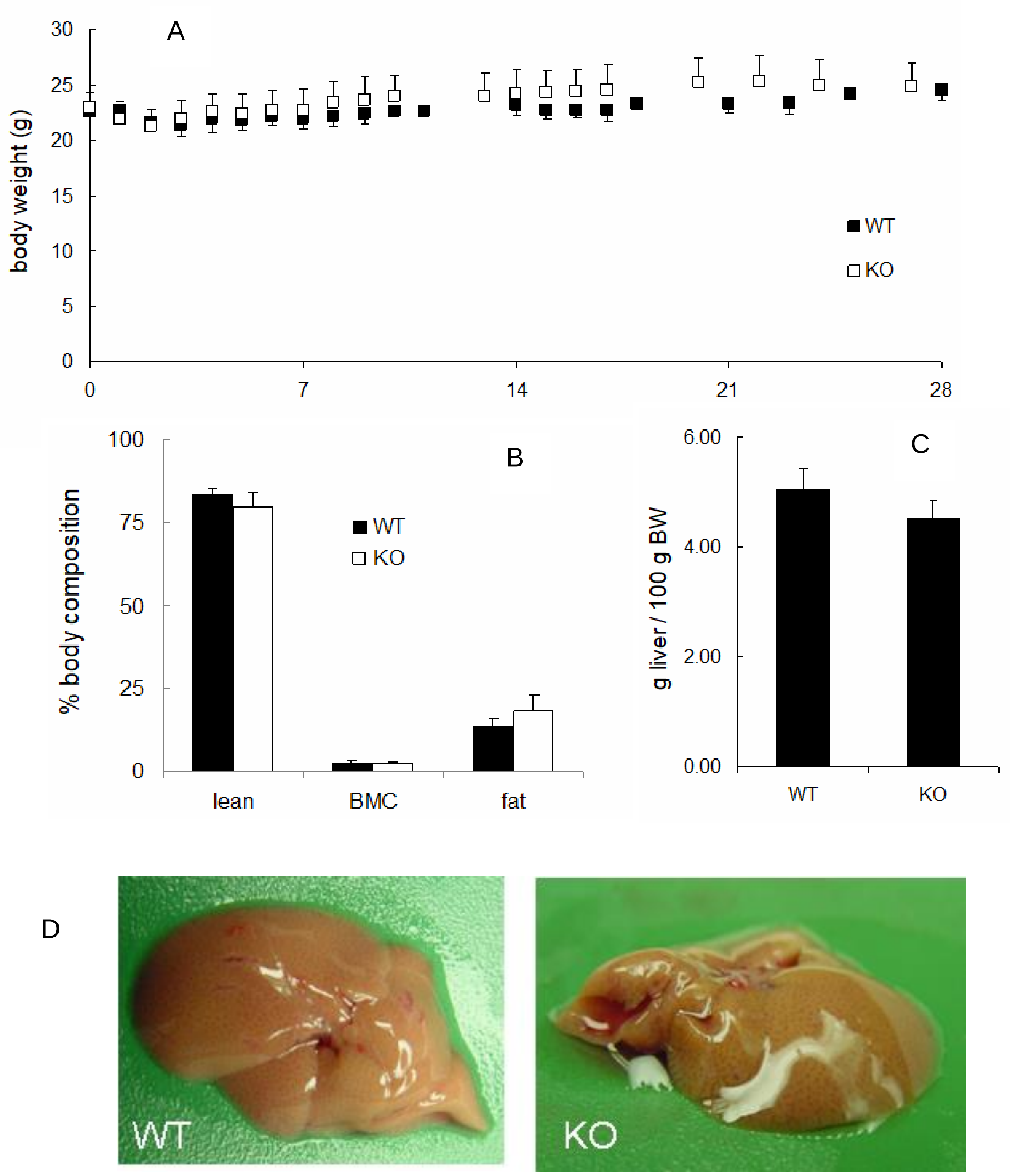

Figure S2-5. Effect of coconut oil enriched high fat diet on PMP34 knockout mice

Adult knockout (KO) or wild type (WT) female littermates were switched from a regular chow to a coconut oil enriched diet at day zero. Body weights were monitored in the forenoon during the following days, but no statistical significant changes were seen, up to 4 weeks of diet (A). DEXA analysis, prior to sacrifice, revealed no difference in body composition (B). Upon dissection, liver size (C) and appearance (D) were similar (with some mottling in both genotypes), as well as abdominal and renal fat deposition (not shown). Values are derived from measurements on four animals per genotype and expressed as mean ± SD. (BMC, bone mineral content).
