# Supplementary material for: Slc25a17 Gene Trapped Mice: PMP34 Plays a Role in the Peroxisomal Degradation of Phytanic and Pristanic Acid
Source: Front Cell Dev Biol. 2020 Mar 24;8:144. doi: 10.3389/fcell.2020.00144 (PMC7106852; doi:10.3389/fcell.2020.00144)
Supplement: Supplementary file 4 [file Data_Sheet_4.pdf]

Supplementary Tables S1-S2.

Table S1. Primers and vectors (relevant restriction sites are underlined)

PCR primers

target	name primer(s)	sequence
Slc25a17 (intron 1)	*Mm*PMP34-in1-s-a	5’-AGCTTGGTAACCAGTGTGG
	*Mm*PMP34-in1-s-b	5’-CTGGCACCTAGCACCTCTTC
	*Mm*PMP34-in1-s-c	5’-TATCCAATGCCCTCTTCTGG
	*Mm*PMP34-in1-s-d	5’-TCCTTCCTGGAACCCTTCTT
	*Mm*PMP34-in1-s-e	5’-CCCCTCCTACAGAGAGACACC
	*Mm*PMP34-in1-s-f	5’-CAAGCTTCCCAGAACAGAGG
	*Mm*PMP34-in1-s-g	5’-GCACAAACACCATGACCAAG
	*Mm*PMP34-in1-s-h	5’-CTTGGCTTTCCAGCTGTAGG
	*Mm*PMP34-in1-s-i	5’-CCAGAGGCTGCTACATTTC
	*Mm*PMP34-in1-s-j	5’-CTTTAGCCTCTGGGCTCTCC
	*Mm*PMP34-in1-s-k	5’-AGGCAGGTTGGGAATTTAGC
	*Mm*PMP34-in1-r-e	5’-CTCTTCCGAAGGTCCTGAGC
βgeo	Gal-s	5’-CGAATACCTGTTCCGTCATAGC
	Gal-r	5’-AGTGGCAACATGGAAATCG
	GAL-3s	5’-CGAATACCTGTTCCGTCATAGCG
	GAL-3r	5’-ACCACTACCATCAATCCGGTAGG
PARP1	HsPARP1-1s-EP	5’-AAGAATTCACTGCAGCTTCTGGAGGACGACAAG
	HsPARP1-2r-XB	5’-AACTCGAGATCTAAAGTTTGGACTTCCACAGGGAGGTCTTAAAAATTG

real time PCR primers

target	name primer(s)	sequence
Slc25a17	PMP34-1	fw 5'-GGCCTCTGTGCTGTCCTACGAAAG; rev 5'- GACTCCGCTCTGGGTGGTAA
Slc25a17	PMP34-2	fw 5'-TGGCACCATGGCCTCTGT; rev 5'-AGTGTGACTGCCATGACAGTGTT

		probe TCCTACGAAAGTCTGGTACACGCCGTGGCCGGAGCCGTG
Cat	Catalase	fw 5'-CGCAGAGACCTGATGTCCTGA; rev 5'- CCCCGCGGTCATGAATATTAA<br>probe 5'-CACCGGAGGCGGGAACCCAATA
Acox1	ACOX1	fw 5'-CACGCACATCTTGGATGGTAGT; rev 5'-TTCGTCAGAATCAAGTTCTCGATT<br>probe 5'-ACACCCGGCGCCGTCGAG
Ehhadh	MFP1	fw 5'-GCTGGAGCCCAGTGACTACCT; rev 5'-CCAAGCTTTGCCATTCTTTCA<br>probe 5'-TGGTTGCCCAGGGAAGCCCTC
Cyp4A10	CytP450A10	fw 5'-GCCAAATCCAGAGGTGTTTGA; rev 5'- AGCAAATTGTTTCCCAATGCA<br>probe 5'-AGACTCTCCCCGACACAGCCACTCATTC
Cpt1β	CPT1β	fw 5'-CTGAGACACATCACCGTCTGGAA; rev 5'- CACCCCTAAGGATGCCATTCT<br>probe 5'- CAACTCCTGGAAGAAACGCCTTATTCGAATC
Actb	actin beta	fw 5'-AGAGGGAAATCGTGCGTGAC; rev 5'-CAATAGTGATGACCTGGCCGT<br>probe 5'-CACTGCCGCATCCTCTTCCTCCC

vectors

**name**	**insert**	**generation, reference or source**
pCR®-XL-TOPO	--	Invitrogen
pCMVβ	β-galactosidase	Clontech
pCMV-Tag2B		Stratagene
pTW45	full length mouse PMP34 cDNA	WylinT, Baes M, Brees C, Mannaerts GP, Fransen M, Van Veldhoven PP (1998) Eur J Biochem 258, 332-338; primer amplified open reading frame inserted into *EcoRI*/*Not*I restricted pBluescript; Wylin T and Van Veldhoven PP, unpublished data.
pRSVL	luciferase	de Wet JR, Wood KV, DeLuca M, Helinski DR, Subramani S. (1987) Mol Cell Biol. 7, 725-737.
pGL3-CMV-luciferase	luciferase	derived from pGL3 (Promega), expression controlled by CMV promotor; Ir. N. Hersmus (Laboratory for Neurobiology, K.U.Leuven)
pRSV-SV40T	SV40 large T antigen	S. Subramani (University of California, San Diego, USA)
pGT1.8IRESβGeo(S)	βgeo	W. Skarnes (Wellcome Trust Sanger Institute, Cambridge, UK)
pMF1706	roGFP-SKL	Ivashchenko O, Van Veldhoven PP, Brees C, Ho YS, Terlecky SR, Fransen M (2011) Mol Biol Cell 22, 1440–1451.

pPVV311	*Hs*PARP$_{511\text{-}1014}$-SKL	this paper

Table S2. Plasmalogen content of tissues of wild type and PMP34 knock out mice.

Plasmalogens were analyzed in lipid extracts of tissues obtained from age matched male adult mice fed a standard (-) or phytol (+) enriched diet, and related to the total amount of phospholipids as described in the Method section. Values are mean ± SD when measurements were performed on tissues derived from two mice, otherwise represent determinations on a single mouse.

.

	plasmalogens (nmol/µmol phospholipid)			
genotype	wild type		PMP34 knock out	
phytol	-	+	-	+
liver	8.97 ± 0.5	9.18 ± 0.4	8.93 ± 0.5	8.30 ± 2.7
cerebrum	229	256 ± 22.0	247	241 ± 12.0
heart	158	123	162	91.6 ± 7.8
